# A tensor-based formulation of hetero-functional graph theory

**DOI:** 10.1038/s41598-022-19333-y

**Published:** 2022-11-05

**Authors:** Amro M. Farid, Dakota J. Thompson, Wester Schoonenberg

**Affiliations:** 1 Thayer School of Engineering at Dartmouth, Hanover, NH USA; 2 MIT Mechanical Engineering, Cambridge, MA USA

**Keywords:** Energy grids and networks, Mechanical engineering

## Abstract

Recently, hetero-functional graph theory (HFGT) has developed as a means to mathematically model the structure of large-scale complex flexible engineering systems. It does so by fusing concepts from network science and model-based systems engineering (MBSE). For the former, it utilizes multiple graph-based data structures to support a matrix-based quantitative analysis. For the latter, HFGT inherits the heterogeneity of conceptual and ontological constructs found in model-based systems engineering including system form, system function, and system concept. These diverse conceptual constructs indicate multi-dimensional rather than two-dimensional relationships. This paper provides the first tensor-based treatment of hetero-functional graph theory. In particular, it addresses the “system concept” and the hetero-functional adjacency matrix from the perspective of tensors and introduces the hetero-functional incidence tensor as a new data structure. The tensor-based formulation described in this work makes a stronger tie between HFGT and its ontological foundations in MBSE. Finally, the tensor-based formulation facilitates several analytical results that provide an understanding of the relationships between HFGT and multi-layer networks.

## Introduction

One defining characteristic of twenty-first century engineering challenges is the breadth of their scope. The National Academy of Engineering (NAE) has identified 14 “game-changing goals”^1^.

1. Advance personalized learning
2. Make solar energy economical
3. Enhance virtual reality
4. Reverse-engineer the brain
5. Engineer better medicines
6. Advance health informatics
7. Restore and improve urban infrastructure
8. Secure cyber-space
9. Provide access to clean water
10. Provide energy from fusion
11. Prevent nuclear terror
12. Manage the nitrogen cycle
13. Develop carbon sequestration methods
14. Engineer the tools of scientific discovery

At first glance, each of these aspirational engineering goals is so large and complex in its own right that it might seem entirely intractable. However, and quite fortunately, the developing consensus across a number of STEM (science, technology, engineering, and mathematics) fields is that each of these goals is characterized by an “engineering system” that is analyzed and re-synthesized using a meta-problem-solving skill set^2^.

### Definition 1

Engineering system^3^: A class of systems characterized by a high degree of technical complexity, social intricacy, and elaborate processes, aimed at fulfilling important functions in society.

**Table 1:** A classification of engineering systems by function and operand^3^.

Function/operand	Living organisms	Matter	Energy	Information	Money
Transform	Hospital	Blast furnace	Engine, electric motor	Analytic engine, calculator	Bureau of printing and engraving
Transport	Car, Airplane, Train	Truck, train, car, airplane	Electricity grid	Cables, radio, telephone, and internet	Banking fedwire and swift transfer systems
Store	Farm, apartment complex	Warehouse	Battery, flywheel, capacitor	Magnetic tape and disk, book	U.S. Buillon repository (Fort Knox)
Exchange	Cattle auction, (illegal) human trafficking	eBay trading system	Energy market	World wide web, wikipedia	London stock exchange
Control	U.S. constitution and laws	National highway traffic safety administration	Nuclear regulatory commission	Internet engineering task force	United States federal reserve

The challenge of ***convergence*** towards ***abstract*** and ***consistent*** methodological foundations for engineering systems is formidable. Consider the engineering systems taxonomy presented in Table 1^3^. It classifies engineering systems by five generic functions that fulfill human needs: (1.) transform (2.) transport (3.) store, (4.) exchange, and (5.) control. On another axis, it classifies them by their operands: (1.) living organisms (including people), (2.) matter, (3.) energy, (4.) information, (5.) money. This classification presents a broad array of application domains that must be consistently treated. Furthermore, these engineering systems are at various stages of development and will continue to be so for decades, if not centuries. And so the study of engineering systems must equally support design synthesis, analysis, and re-synthesis while supporting innovation; be it incremental or disruptive.

### Background literature

Two fields in particular have attempted to traverse this convergence challenge: systems engineering and network science. Systems engineering, and more recently model-based systems engineering (MBSE), has developed as a practical and interdisciplinary engineering discipline that enables the successful realization of complex systems from concept, through design, to full implementation^4^. It equips the engineer with methods and tools to handle systems of ever-greater complexity arising from greater interactions within these systems or from the expanding heterogeneity they demonstrate in their structure and function. Despite its many accomplishments, model-based systems engineering still relies on graphical modeling languages that provide limited quantitative insight (on their own)^5–7^.

In contrast, network science has developed to quantitatively analyze networks that appear in a wide variety of engineering systems. And yet, despite its methodological developments in multi-layer networks, network science has often been unable to address the explicit heterogeneity often encountered in engineering systems^7,8^. In a recent comprehensive review Kivela et. al^8^ write:

“The study of multi-layer networks $\ldots$ has become extremely popular. Most real and engineered systems include multiple subsystems and layers of connectivity and developing a deep understanding of multi-layer systems necessitates generalizing ‘traditional’ graph theory. Ignoring such information can yield misleading results, so new tools need to be developed. One can have a lot of fun studying ‘bigger and better’ versions of the diagnostics, models and dynamical processes that we know and presumably love – and it is very important to do so but the new ‘degrees of freedom’ in multi-layer systems also yield new phenomena that cannot occur in single-layer systems. Moreover, the increasing availability of empirical data for fundamentally multi-layer systems amidst the current data deluge also makes it possible to develop and validate increasingly general frameworks for the study of networks.

$\ldots$ Numerous similar ideas have been developed in parallel, and the literature on multi-layer networks has rapidly become extremely messy. Despite a wealth of antecedent ideas in subjects like sociology and engineering, many aspects of the theory of multi-layer networks remain immature, and the rapid onslaught of papers on various types of multilayer networks necessitates an attempt to unify the various disparate threads and to discern their similarities and differences in as precise a manner as possible.

$\ldots$ [The multi-layer network community] has produced an equally immense explosion of disparate terminology, and the lack of consensus (or even generally accepted) set of terminology and mathematical framework for studying is extremely problematic.”

In many ways, the parallel developments of the model-based systems engineering and network science communities intellectually converge in *hetero-functional graph theory* (HFGT)^7^. For the former, it utilizes multiple graph-based data structures to support a matrix-based quantitative analysis. For the latter, HFGT inherits the heterogeneity of conceptual and ontological constructs found in model-based systems engineering including system form, system function, and system concept. More specifically, the explicit treatment of function and operand facilitates a structural understanding of the diversity of engineering systems found in Table 1. Although not named as such originally, the first works on HFGT appeared as early as 2006–2008^9–12^. Since then, HFGT has become multiply established and demonstrated cross-domain applicability^7,13^; culminating in the recent consolidating text^7^.

The primary benefit of HFGT, relative to multi-layer networks, is the broad extent of its ontological elements and associated mathematical models^7^. In their recent review, Kivela et. al showed that *all* of the reviewed works have exhibited at least one of the following modeling constraints^8^:

1. Alignment of nodes between layers is required^8,14–62^
2. Disjointment between layers is required^8,51,56,63–80^
3. Equal number of nodes for all layers is required^8,14–51,59,61,65,67,71,72,81,82^
4. Exclusively vertical coupling between all layers is required^8,14–51,59,61,66,82–86^
5. Equal couplings between all layers are required^8,16–41,45–51,59,61,66,82–86^
6. Node counterparts are coupled between all layers^16–20,24–41,45–51,59,61,66,82–86^
7. Limited number of modelled layers^47–49,51,56,59,61,63–93^
8. Limited number of aspects in a layer^14–51,56,59,61,63–80,82^

To demonstrate the consequences of these modeling limitations, the HFGT text^7^ developed a very small, but highly heterogeneous, hypothetical test case system that exhibited *all eight* of the modeling limitations identified by Kivela et. al. Consequently, none of the multi-layer network models identified by Kivela et. al. would be able to model such a hypothetical test case. In contrast, a complete HFGT analysis of this hypothetical test case was demonstrated in the aforementioned text^7^.

**Figure 1:**
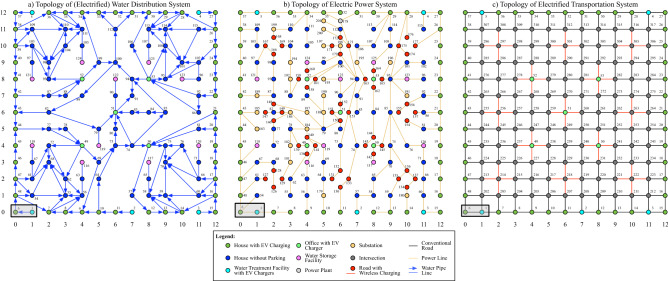
A topological visualizaiton of the trimetrica smart city infrastructure test case^7^.

The same text provides the even more complex hypothetical smart city infrastructure example shown in Fig. 1. It not only includes an electric power system, water distribution system, and electrified transportation system but it also makes very fine distinctions in the functionality of its component elements.

**Figure 2:**
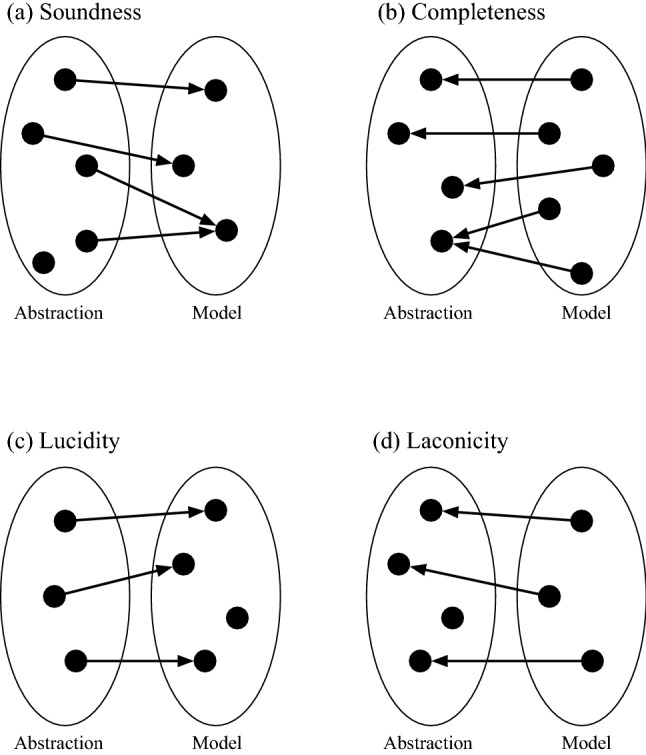
Graphical representation of four ontological properties as mapping between abstraction and model: (**a**) soundness, (**b**) completeness, (**c**) lucidity, and (**d**) laconicity^95^.

Given the quickly developing *“disparate terminology and the lack of consensus”*, Kivela et. al.’s^8^ stated goal *“to unify the various disparate threads and to discern their similarities and differences in as precise a manner as possible”* appears imperative. While many may think that the development of mathematical models is subjective, in reality, ontological science presents a robust methodological foundation. As briefly explained in Online Appendix A, and as detailed elsewhere^7,94,95^, the process of developing a mathematical model of a given (engineering) system is never direct. Rather, a specific engineering system (which is an instance of a class of systems) has abstract elements in the mind (It is likely that modeling abstract elements in the mind is unfamiliar to this journal’s readership. This is purely an issue of nomenclature. Most physicists and engineers would agree on the indispensable role that *intuition* – itself a mental model – has to the development of mathematical models of systems. For example, the shift from Newtonian mechanics to Einstein’s relativity constituted first an expansion in the abstract elements of the mental model and their relationships well before that mental model could be translated into its associated mathematics. Similarly, the “disparate terminology and lack of consensus” identified by Kivela et. al^8^ suggests that a reconciliation of this abstract mental model is required (see “Background literature”) that constitute an *abstraction* ${\mathcal {A}}$ (which is an instance of a domain conceptualization ${{\mathcal {C}}}$). ${{\mathcal {C}}}$ is mapped to a set of primitive mathematical elements called a language ${\mathcal {L}}$, which is in turn instantiated to produce a mathematical model ${{\mathcal {M}}}$. The fidelity of the mathematical model with respect to an abstraction is determined by the four complementary linguistic properties shown in Fig. 2^95^: soundness, completeness, lucidity, and laconicity^96^ (See Appendix Defns. 1–4). When all four properties are met, the abstraction and the mathematical model have an isomorphic (one-to-one) mapping and faithfully represent each other. For example, the network science and graph theory literature assume an abstract conceptualization of nodes and edges prior to defining their 1-to-1 mathematical counterparts^7^. Consequently, as hetero-functional graph and multi-layer network models of engineering systems are developed, there is a need to reconcile both the abstraction and the mathematical model on the basis of the four criteria identified above (See Appendix A.).

The ontological strength of hetero-functional graph theory comes from the “systems thinking” foundations in the model-based systems engineering literature^7,97^. In effect, and very briefly, all systems have a “subject + verb + operand” form where the system form is the subject, the system function is the verb + operand (i.e. predicate) and the system concept is the mapping of the two to each other. The key distinguishing feature of HFGT (relative to multi-layer networks) is its introduction of system function. In that regard, it is more complete than multi-layer networks if system function is accepted as part of an engineering system abstraction. Another key distinguishing feature of HFGT is the differentiation between elements related to transformation and transportation. In that regard, it takes great care to not *overload* mathematical modeling elements and preserve lucidity.

### Original contribution

This paper provides a tensor-based formulation of several of the most important parts of hetero-functional graph theory. More specifically, it discusses the system concept, the hetero-functional adjacency matrix, and the hetero-functional incidence tensor. Whereas the hetero-functional graph theory text^7^ is a comprehensive discussion of the subject, the treatment is based entirely on two-dimensional matrices. The tensor-based formulation described in this work makes a stronger tie between HFGT and its ontological foundations in MBSE. Furthermore, the tensor-based treatment developed here reveals patterns of underlying structure in engineering systems that are less apparent in a matrix-based treatment. Finally, the tensor-based formulation facilitates an understanding of the relationships between HFGT and multi-layer networks (“despite its disparate terminology and lack of consensus”). In so doing, this tensor-based treatment is likely to advance Kivela et. al’s goal to discern the similarities and differences between these mathematical models in as precise a manner as possible.

### Paper outline

The rest of the paper is organized as follows. “The system concept” discusses the system concept as an allocation of system function to system form. “Hetero-functional adjacency matrix” discusses the hetero-functional adjacency matrix emphasizing the relationships between system capabilities (i.e. structural degrees of freedom as defined therein). “Hetero-functional incidence tensor”, then, discusses the hetero-functional incidence tensor which describes the relationships between system capabilities, operands, and physical locations in space (i.e. system buffers as defined later). “Discussion” goes on to discuss this tensor-based formulation from the perspective of layers and network descriptors. “Conclusions and future work” brings the work to a close and offers directions for future work. Given the multi-disciplinary nature of this work, several appendices are provided to support the work with background material and avoid breaking the logical flow of the main article. Appendix A provides the fundamental definitions of ontological science that were used to motivate this work’s original contribution. Appendix B describes the notation conventions used throughout this work. The paper assumes that the reader is well grounded in graph theory and network science as it is found in any one of a number of excellent texts^98,99^. The paper does not assume prior exposure to hetero-functional graph theory. It’s most critical definitions are tersely introduced in the body of the work upon first mention. More detailed classifications of these concepts are compiled in Appendix C for convenience. Given the theoretical treatment provided here, the interested reader is referred to the hetero-functional graph theory text^7^ for further explanation of these well-established concepts and concrete examples. Furthermore, several recent works have made illustrative comparisons between (formal) graphs and hetero-functional graphs^100,101^. Finally, this work makes extensive use of set, Boolean, matrix, and tensor operations; all of which are defined unambiguously in Appendices D, E, F, and G respectively.

## The system concept

At a high-level, the system concept $A_S$ describes the allocation of system function to system form as the central question of engineering design. First, “System resources, processes, and knowledge base” provides introductory definitions of system resources, processes, and knowledge base that serve as prerequisite knowledge for the remaining subsections. Next, “The transportation knowledge base tensor” introduces the transportation knowledge base as a third-order tensor. Next, “The refined transportation knowledge base tensor” introduces the refined transportation knowledge base as a fourth-order tensor. The tensor-based formulations in these two subsection directly support the original contribution mentioned in “Original contribution”. Finally, in order to support the following section, “Existence, availability, and concept of system capabilities” concludes the section with a discussion of existence and availability of system capabilities as part of the system concept.

### System resources, processes, and knowledge base

This dichotomy of form and function is repeatedly emphasized in the fields of engineering design and systems engineering^97,102–104^. More specifically, the allocation of system processes to system resources is captured in the “design equation”^10,94^:

1 $$\begin{aligned} P=J_S\odot R \end{aligned}$$

where *R* is set of system resources, *P* is the set of system processes, $J_S$ is the system knowledge base, and $\odot$ is matrix Boolean multiplication (Online Appendix Defn. 24).

#### Definition 2

(*System Resource*^4^) An asset or object $r_v \in R$ that is utilized during the execution of a process.

#### Definition 3

(*System Process*^4,105^) An activity $p \in P$ that transforms a predefined set of input operands into a predefined set of outputs.

#### Definition 4

(*System Operand*^4^) An asset or object $l_i \in L$ that is operated on or consumed during the execution of a process.

#### Definition 5

(*System Knowledge Base*^9–13,94^) A binary matrix $J_S$ of size $\sigma (P)\times \sigma (R)$ whose element $J_S(w,v)\in \{0,1\}$ is equal to one when action $e_{wv} \in {{\mathcal {E}}}_S$ (in the SysML sense) exists as a system process $p_w \in P$ being executed by a resource $r_v \in R$. The $\sigma ()$ notation gives the size of a set.

In other words, the system knowledge base forms a bipartite graph between the set of system processes and the set of system resources^13^.

Hetero-functional graph theory further recognizes that there are inherent differences within the set of resources as well as within the set of processes. Therefore, classifications of these sets of resources and sets of processes are introduced and defined in Appendix C. $R=M \cup B \cup H$ where *M* is the set of transformation resources (Definition 5), *B* is the set of independent buffers (Definition 6), and *H* is the set of transportation resources (Definition 7). Furthermore, the set of buffers $B_S=M \cup B$ (Definition 8) is introduced for later discussion. Similarly, $P = P_\mu \cup P_{\bar{\eta }}$ where $P_\mu$ is the set of transformation processes (Definition 9) and $P_{\bar{\eta }}$ is the set of refined transportation processes (Definition 10). The latter, in turn, is determined from the Cartesian product ($\times$) (Definition 21) of the set of transportation processes $P_\eta$ (Definition 11) and the set of holding processes $P_\gamma$ (Definition 12).

2 $$\begin{aligned} P_{\bar{\eta }} = P_{\gamma } {\times } P_{\eta } \end{aligned}$$

**Figure 3:**
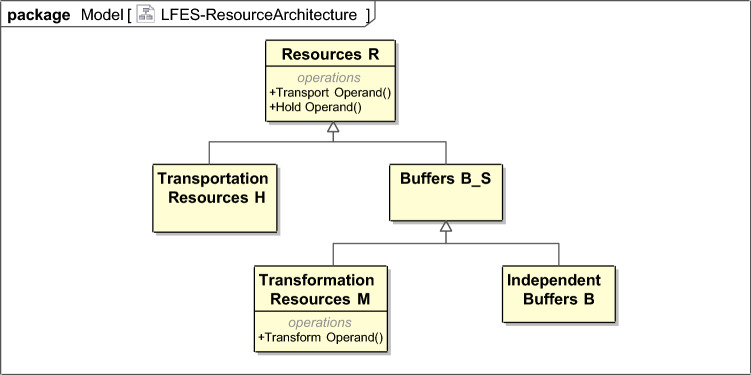
The hetero-functional graph theory meta-architecture drawn using the systems markup language (SysML). It consists of three types of resources $R = M \cup B \cup H$ that are capable of two types of process $P_{\bar{\eta }} = P_{\gamma } {\times } P_{\eta }$^7^.

This taxonomy of resources, processes, and their allocation is organized in the HFGT meta-architecture shown in Fig. 3. The taxonomy of resources *R* and processes *P* originates from the field of production systems where transformation processes are viewed as “value-adding”, holding processes support the design of fixtures, and transportation processes are cost-minimized. Furthermore, their existence is necessitated by their distinct roles in the structural relationships found in hetero-functional graphs. Consequently, subsets of the design Eq. (1) can be written to emphasize the relationships between the constitutent classes of processes and resources^9–13^.

3 $$\begin{aligned} P_\mu&= J_M\odot M \end{aligned}$$

4 $$\begin{aligned} P_\gamma&= J_\gamma \odot R \end{aligned}$$

5 $$\begin{aligned} P_\eta&= J_H\odot R \end{aligned}$$

6 $$\begin{aligned} P_{\bar{\eta }}&= J_{\bar{H}} \odot R \end{aligned}$$

where $J_M$ is the transformation knowledge base, $J_\gamma$ is the holding knowledge base, $J_H$ is the transportation knowledge base, and $J_{\bar{H}}$ is the refined transportation knowledge base^10,13,106–109^. The original system knowledge base $J_S$ is straightforwardly reconstructed from these smaller knowledge bases^9–13^:

7 $$\begin{aligned} J_{S}= \left[ \begin{array}{ccc} J_{M}&{}|&{}{\mathbf {0}}\\ \hline &{}J_{\bar{H}}&{}\\ \end{array}\right] \end{aligned}$$

### The transportation knowledge base tensor

The transportation knowledge base $J_H$ is best understood as a *matricized* 3rd-order tensor ${{\mathcal {J}}}_H$ where the element ${{\mathcal {J}}}_H(y_1,y_2,v)=1$ when the transportation process $p_u \in P_\eta$ defined by the origin $b_{s_{y_1}} \in B_{S}$ and the destination $b_{s_{y_2}} \in B_{S}$ is executed by the resource $r_v \in R$.

8 $$\begin{aligned} J_H&={{\mathcal {F}}}_M\left( {{\mathcal {J}}}_H,[2,1],[3]\right) \end{aligned}$$

9 $$\begin{aligned} {{\mathcal {J}}}_H&={{\mathcal {F}}}_M^{-1}\left( J_H,[\sigma (B_S),\sigma (B_S),\sigma (R)],[2,1],[3]\right) \end{aligned}$$

where ${{\mathcal {F}}}_M$ and ${{\mathcal {F}}}_M^{-1}$ are the matricization and tensorization functions (Defns. 33 and 34) respectively. Here, ${{\mathcal {F}}}_M()$ serves to vectorize the dimensions of the origin and destination buffers into the single dimension of transportation processes. Appendix G, more generally, introduces the reader to tensor-based operations.

The ${{\mathcal {J}}}_H$ tensor reveals that the transportation knowledge base is closely tied to the classical understanding of a graph $A_{B_S}$ where point elements of form called nodes, herein taken to be the set of buffers $B_S$, are connected by line elements of form called edges. Such a graph in hetero-functional graph theory (and model-based systems engineering) is called a formal graph^97^ because all of its elements describe the system form and any statement of function is entirely *implicit*.

10 $$\begin{aligned} A_{B_S}(y_1,y_2)&=\bigvee _v^{\sigma (R)}{{\mathcal {J}}}_H(y_1,y_2,v)\nonumber \\&= \bigvee _v^{\sigma (R)}J_H(u,v) \qquad \forall y_1,y_2 \in \{1,\ldots , \sigma (B_S)\} , u = \sigma (B_S)(y_1 -1) + y_2, v \in \{1,\ldots , \sigma (R)\} \end{aligned}$$

11 $$\begin{aligned} A_{B_S}&= {{\mathcal {J}}}_H \odot _3 {\mathbbm {1}}^{\sigma (R)} =vec^{-1}\left( J_H\odot {\mathbbm {1}}^{\sigma (R)},[\sigma (B_S),\sigma (B_S)]\right) ^T \end{aligned}$$

12 $$\begin{aligned} A_{B_S}^{TV}&= \left( {{\mathcal {J}}}_H \odot _3 {\mathbbm {1}}^{\sigma (R)}\right) ^{TV} =J_H\odot \mathbbm {1}^{\sigma (R)} \end{aligned}$$

where the $\bigvee$ notation is the Boolean analogue of the $\sum$ notation (Definition 22), $\odot _n$ is the n-mode Boolean matrix product (Online Appendix Defn. 39), $vec^{-1}()$ is inverse vectorization (Online Appendix Defn. 36) and $()^V$ is shorthand for vectorization (Online Appendix Defn. 35). Appendix E, more generally, introduces the reader to Boolean operations. Furthermore, the notation $\mathbbm {1}^n$ is used to indicate a ones-vector of length n. The transportation system knowledge base $J_H$ replaces the edges of the formal graph $A_{B_S}$ with an *explicit* description of function in the transportation processes $P_\eta$. The multi-column nature of the transportation knowledge base $J_H$ contains more information than the formal graph $A_{B_S}$ and allows potentially many resources to execute any given transportation process. Consequently, the OR operation across the rows of $J_H$ (or the third dimension of ${{\mathcal {J}}}_H$) is sufficient to reconstruct the formal graph $A_{B_S}$. In short, a single column transportation knowledge base is mathematically equivalent to a vectorized formal graph $A_{B_S}$.

### The refined transportation knowledge base tensor

Similarly, the refined transportation knowledge base is best understood as a matricized 4th order tensor ${{\mathcal {J}}}_{\bar{H}}$ where the element ${{\mathcal {J}}}_H(g,y_1,y_2,v)=1$ when the refined transportation process $p_\varphi \in P_{\bar{\eta }}$ defined by the holding process $p_{\gamma g} \in P_\gamma$, the origin $b_{s_{y_1}} \in B_{S}$ and the destination $b_{s_{y_2}} \in B_{S}$ is executed by the resource $r_v \in R$.

13 $$\begin{aligned} J_{\bar{H}}&={{\mathcal {F}}}_M\left( {{\mathcal {J}}}_{\bar{H}},[3,2,1],[4]\right) \end{aligned}$$

14 $$\begin{aligned} {{\mathcal {J}}}_{\bar{H}}&={{\mathcal {F}}}_M^{-1}\left( J_{\bar{H}},[\sigma (P_\gamma ),\sigma (B_S),\sigma (B_S),\sigma (R)],[3,2,1],[4]\right) \end{aligned}$$

The $J_{\bar{H}}$ tensor reveals that the refined transportation knowledge base is closely tied to the classical understanding of a multi-commodity flow network ${{\mathcal {A}}}_{L B_S}$^110–112^. Mathematically, it is a 3rd-order tensor whose element ${{\mathcal {A}}}_{L B_S}(i,y_1,y_2)=1$ when operand $l_i \in L$ is transported from buffer $b_{s_{y_1}}$ to $b_{s_{y_2}}$. Again, the multi-commodity flow network ${{\mathcal {A}}}_{L B_S}$ is purely a description of system form and any statement of function is entirely *implicit*. In the special case (By Definition 12, holding processes are distinguished by three criteria: 1.) different operands, 2.) how they hold those operands, and 3.) if they change the state of the operand. The special case mentioned above is restricted to only the first of these three conditions.) of a system where the set of operands *L* maps 1-to-1 the set of holding processes $P_\gamma$ (i.e. $i=g$):

15 $$\begin{aligned} {{\mathcal {A}}}_{L B_S}(i,y_1,y_2)&=\bigvee _v^{\sigma (R)}{{\mathcal {J}}}_{\bar{H}}(g,y_1,y_2,v) \quad \forall g \in \{1,\ldots , \sigma (P_\gamma )\} , y_1,y_2 \in \{1,\ldots , \sigma (B_S)\} , v \in \{1,\ldots , \sigma (R)\} \end{aligned}$$

16 $$\begin{aligned}&= \bigvee _v^{\sigma (R)}J_{\bar{H}}(\varphi ,v) \quad \forall \varphi =\sigma ^2(B_S)(g-1)+\sigma (B_S)(y_1-1)+y_2, v \in \{1,\ldots , \sigma (R)\} \end{aligned}$$

17 $$\begin{aligned} {{\mathcal {A}}}_{L B_S}&= {{\mathcal {J}}}_{\bar{H}} \odot _4 \mathbbm {1}^{\sigma (R)} =vec^{-1}\left( J_{\bar{H}}\odot \mathbbm {1}^{\sigma (R)},\left[ \sigma (B_S),\sigma (B_S),\sigma (P_\gamma )\right] \right) ^T \end{aligned}$$

18 $$\begin{aligned} {{\mathcal {A}}}_{L B_S}^{TV}&= \left( {{\mathcal {J}}}_{\bar{H}} \odot _4 \mathbbm {1}^{\sigma (R)}\right) ^{TV} =J_{\bar{H}}\odot \mathbbm {1}^{\sigma (R)} \end{aligned}$$

The refined transportation system knowledge base $J_{\bar{H}}$ replaces the operands and edges of the multi-commodity flow network $A_{MP}$ with an *explicit* description of function in the holding processes $P_\gamma$ and transportation processes $P_\eta$. The multi-column nature of the refined transportation knowledge base $J_{\bar{H}}$ contains more information than the multi-commodity flow network ${{\mathcal {A}}}_{L B_S}$ and allows potentially many resources to execute any given refined transportation process. Consequently, the OR operation across the rows of $J_{\bar{H}}$ (or the fourth dimension of ${{\mathcal {J}}}_{\bar{H}}$) is sufficient to reconstruct the multi-commodity flow network ${{\mathcal {A}}}_{L B_S}$. In short, a single column of the refined transportation knowledge base is mathematically equivalent to a vectorized multi-commodity flow network.

The transformation, holding, transportation and refined transportation knowledge bases ($J_M$, $J_\gamma$, $J_H$ and $J_{\bar{H}}$) readily serve to reconstruct the system knowledge base $J_S$. First, the refined transportation knowledge base is the Khatri-Rao product of the holding and transportation knowledge bases.

19 $$\begin{aligned} J_{\bar{H}}(\sigma (P_\eta )(g-1)+u,v)&=J_\gamma (g,v) \cdot J_H(u,v)\nonumber \\&\quad \forall g \in \{1,\ldots , \sigma (P_\gamma )\} , u = \sigma (B_S)(y_1 -1) + y_2, v \in \{1,\ldots , \sigma (R)\} \end{aligned}$$

20 $$\begin{aligned} J_{\bar{H}}&=J_\gamma \circledast J_H \end{aligned}$$

21 $$\begin{aligned}&= \left[ J_\gamma \otimes \mathbbm {1}^{\sigma (P_\eta )}\right] \cdot \left[ \mathbbm {1}^{\sigma (P_\gamma )} \otimes J_H \right] \end{aligned}$$

where $\cdot$ is the Hadamard (or scalar) product (Online Appendix Defn. 28), $\circledast$ is the Khatri-Rao product (Online Appendix Defn. 31) and $\otimes$ is the Kronecker product (Online Appendix Defn. 30).

### Existence, availability, and concept of system capabilities

Hetero-functional graph theory also differentiates between the *existence* and the *availability* of physical capabilities in the system^10,107^. While the former is described by the system knowledge base the latter is captured by the system constraints matrix (which is assumed to evolve in time).

#### Definition 6

(*System Constraints Matrix*^9–13,94^) A binary matrix $K_S$ of size $\sigma (P)\times \sigma (R)$ whose element $K_S(w,v)\in \{0,1\}$ is equal to one when a constraint eliminates event $e_{wv}$ from the event set.

The system constraints matrix is constructed analogously to the system knowledge base^9–13^.

22 $$\begin{aligned} K_{S}= \left[ \begin{array}{ccc} K_{M}&{}|&{}{\mathbf {0}}\\ \hline &{}K_{\bar{H}}&{}\\ \end{array}\right] \end{aligned}$$

In this regard, the system constraints matrix has a similar meaning to graph percolation^113,114^ and temporal networks^115^.

Once the system knowledge base $J_S$ and the system constraints matrix $K_S$ have been constructed, the system concept $A_S$ follows straightforwardly.

#### Definition 7

(*System Concept*^9–13,94^) A binary matrix $A_S$ of size $\sigma (P)\times \sigma (R)$ whose element $A_S(w,v)\in \{0,1\}$ is equal to one when action $e_{wv} \in {{\mathcal {E}}}_S$ (in the SysML sense) is available as a system process $p_w \in P$ being executed by a resource $r_v \in R$.

23 $$\begin{aligned} A_S=J_S\ominus K_S=J_S\cdot \bar{K}_S \end{aligned}$$

where $\ominus$ is Boolean subtraction (Online Appendix Defn. 26) and ${\overline{K}}_S=NOT(K_S)$.

Every filled element of the system concept indicates a *system capability* (Definition 13) of the form: “Resource $r_v$ does process $p_w$”. The system constraints matrix limits the availability of capabilities in the system knowledge base to create the system concept $A_S$. The system capabilities are quantified by the structural degrees of freedom.

#### Definition 8

(*Structural Degrees of Freedom*^9–13,94^) The set of independent actions ${{\mathcal {E}}}_S$ that completely defines the instantiated processes in a large flexible engineering system. Their number is given by:

24 $$\begin{aligned} DOF_S=\sigma ({{\mathcal {E}}}_S)&=\sum _w^{\sigma (P)}\sum _v^{\sigma (R)}\left[ J_S\ominus K_S \right] (w,v) \end{aligned}$$

25 $$\begin{aligned}&=\sum _w^{\sigma (P)}\sum _v^{\sigma (R)}A_S(w,v) \end{aligned}$$

26 $$\begin{aligned}&= \langle J_S, \bar{K}_S \rangle _F \end{aligned}$$

As has been discussed extensively in prior publications, the term structural degrees of freedom is best viewed as a generalization of kinematic degrees of freedom (or generalized coordinates)^9–13,116^. Note that the transformation degrees of freedom $DOF_M$ and the refined transportation degrees of freedom $DOF_H$ are calculated similarly^9–12^:

27 $$\begin{aligned} DOF_M&= \sum _j^{\sigma (P_\mu )}\sum _k^{\sigma (M)}\left[ J_M\ominus K_M \right] (j,k) \end{aligned}$$

28 $$\begin{aligned} DOF_H&= \sum _\varphi ^{\sigma (P_{\bar{\eta }})}\sum _v^{\sigma (R)}\left[ J_{\bar{H}}\ominus K_{\bar{H}} \right] (u,v) \end{aligned}$$

## Hetero-functional adjacency matrix

This section serves provides a tensor-based formulation of the hetero-functional adjacency matrix. First, “Pairwise sequences of system capabilities” introduces this matrix as pairwise sequences of system capabilities. Next, “The system sequence knowledge base tensor” provides a tensor-based formulation of the system sequence knowledge base. Next, “The system sequence constraints tensor” provides a tensor-based formulation of the system sequence constraints. Both of these subsections directly support the paper’s original contribution. Finally, “Sequence-dependent degrees of freedom” concludes the section with a discussion of sequence-dependent degrees of freedom.

### Pairwise sequences of system capabilities

Once the system’s physical capabilities (or structural degrees of freedom have been defined), the hetero-functional adjacency matrix $A_\rho$ is introduced to represent their pairwise sequences^13,108,117–119^.

#### Definition 9

(*Hetero-functional Adjacency Matrix*^13,108,117–119^) A square binary matrix $A_\rho$ of size $\sigma (R)\sigma (P)\times \sigma (R)\sigma (P)$ whose element $J_\rho (\chi _1,\chi _2)\in \{0,1\}$ is equal to one when string $z_{\chi _1,\chi _2}=e_{w_1v_1}e_{w_2v_2} \in {{\mathcal {Z}}}$ is available and exists, where index $\chi _i \in \left[ 1, \dots ,\sigma (R)\sigma (P)\right]$.

In other words, the hetero-functional adjacency matrix corresponds to a hetero-functional graph $G = \{{{\mathcal {E}}}_S, {{\mathcal {Z}}} \}$ with structural degrees of freedom (i.e. capabilities) ${{\mathcal {E}}}_S$ as nodes and feasible sequences ${{\mathcal {Z}}}$ as edges.

Much like the system concept $A_S$, the hetero-functional adjacency matrix $A_\rho$ arises from a Boolean difference^13,108,117–119^.

29 $$\begin{aligned} A_{\rho } = J_{\rho } \ominus K_\rho \end{aligned}$$

where $J_\rho$ is the system sequence knowledge base and $K_\rho$ is the system sequence constraints matrix.

#### Definition 10

(*System Sequence Knowledge Base*^13,108,117–119^) A square binary matrix $J_\rho$ of size $\sigma (R)\sigma (P)\times \sigma (R)\sigma (P)$ whose element $J_\rho (\chi _1,\chi _2)\in \{0,1\}$ is equal to one when string $z_{\chi _1,\chi _2}=e_{w_1v_1}e_{w_2v_2} \in {{\mathcal {Z}}}$ exists, where index $\chi _i \in \left[ 1, \dots ,\sigma (R)\sigma (P)\right]$.

#### Definition 11

(*System Sequence Constraints Matrix*^13,108,117–119^) A square binary constraints matrix $K_{\rho }$ of size $\sigma (R)\sigma (P)\times \sigma (R)\sigma (P)$ whose elements $K(\chi _1,\chi _2)\in \{0,1\}$ are equal to one when string $z_{\chi _1 \chi _2}=e_{w_1v_1}e_{w_2v_2} \in {{\mathcal {Z}}}$ is eliminated.

The definitions of the system sequence knowledge base $J_\rho$ and the system sequence constraints matrix $K_\rho$ feature a translation of indices from $e_{w_1v_1}e_{w_2v_2}$ to $z_{\chi _1 \chi _2}$. This fact suggests that these matrices have their associated 4th order tensors ${{\mathcal {J}}}_\rho$, ${{\mathcal {K}}}_\rho$ and ${{\mathcal {A}}}_\rho$.

30 $$\begin{aligned} J_\rho&={{\mathcal {F}}}_M\left( {{\mathcal {J}}}_\rho ,[1,2],[3,4]\right) \end{aligned}$$

31 $$\begin{aligned} K_\rho&={{\mathcal {F}}}_M\left( {{\mathcal {K}}}_\rho ,[1,2],[3,4]\right) \end{aligned}$$

32 $$\begin{aligned} A_\rho&={{\mathcal {F}}}_M\left( {{\mathcal {A}}}_\rho ,[1,2],[3,4]\right) \end{aligned}$$

### The system sequence knowledge base tensor

The system sequence knowledge base $J_\rho$ and its tensor-equivalent ${{\mathcal {J}}}_\rho$ create all the *potential* sequences of the capabilities in $A_S$.

33 $$\begin{aligned} {{\mathcal {J}}}_\rho (w_1,v_1,w_2,v_2)&=A_S(w_1,v_1)\cdot A_S(w_2,v_2) \quad \forall w_1,w_2 \in \{1\ldots \sigma (P)\} , v_1,v_2 \in \{1\ldots \sigma (R)\} \end{aligned}$$

34 $$\begin{aligned} {J}_\rho (\chi _1,\chi _2)&=A_S^V(\chi _1)\cdot A^V_S(\chi _2) \qquad \quad \;\; \forall \chi _1,\chi _2 \in \{1\ldots \sigma (R)\sigma (P)\} \end{aligned}$$

35 $$\begin{aligned} J_\rho&=A_S^VA_S^{VT} \end{aligned}$$

36 $$\begin{aligned} J_\rho&=\left[ J_S\cdot \bar{K}_S\right] ^V\left[ J_S\cdot \bar{K}_S\right] ^{VT} \end{aligned}$$

37 $$\begin{aligned} {{\mathcal {J}}}_\rho&={{\mathcal {F}}}_M^{-1}\left( J_\rho ,[\sigma (P),\sigma (R),\sigma (P),\sigma (R)],[1,2],[3,4]\right) \end{aligned}$$

### The system sequence constraints tensor

Of these potential sequences of capabilities, the system sequence constraints matrix $K_\rho$ serves to eliminate the infeasible pairs. The feasibility arises from five types of constraints:

I: $P_\mu P_\mu$. Two transformation processes that follow each other must occur at the same transformation resource. $m_1=m_2$.
II: $P_\mu P_{\bar{\eta }}$. A refined transportation process that follows a transformation process must have an origin equivalent to the transformation resource at which the transformation process was executed. $m_1-1=(u_1-1)/\sigma (B_S)$ where / indicates integer division.
III: $P_{\bar{\eta }} P_\mu$. A refined transportation process that precedes a transformation process must have a destination equivalent to the transformation resource at which the transformation process was executed. $m_2-1= (u_1-1)\%\sigma (B_S)$ where $\%$ indicates the modulus.
IV: $P_{\bar{\eta }} P_{\bar{\eta }}$. A refined transportation process that follows another must have an origin equivalent to the destination of the other. $(u_1-1) \% \sigma (B_S) = (u_2-1)/\sigma (B_S)$
V: *PP*. The type of operand of one process must be equivalent to the type of output of another process. In other words, the ordered pair of processes $P_{w_1}P_{w_2}$ is feasible if and only if $A_P(w_1,w_2)=1$ where $A_P$ is the adjacency matrix that corresponds to a *functional graph* in which pairs of system processes are connected.

In previous hetero-functional graph theory works, the system sequence constraints matrix $K_\rho$ was calculated straightforwardly using for FOR loops to loop over the indices $\chi _1$ and $\chi _2$ and checking the presence of the five feasibility constraints identified above.

Here, an alternate approach based upon tensors is provided for insight into the underlying mathematical structure. For convenience, ${\overline{K}}_\rho =NOT(K_\rho )$ captures the set of all *feasibility* conditions that pertain to valid sequences of system capabilities. This set requires that *any* of the first four constraints above *and* the last constraint be satisfied.

38 $$\begin{aligned} {\overline{K}}_\rho =\left( {\overline{K}}_{\rho I} \oplus {\overline{K}}_{\rho II} \oplus {\overline{K}}_{\rho III} \oplus {\overline{K}}_{\rho IV} \right) \cdot {\overline{K}}_{\rho V} \end{aligned}$$

where $\oplus$ is Boolean addition (Definition 23) and ${\overline{K}}_{\rho I}, {\overline{K}}_{\rho II}, {\overline{K}}_{\rho III}, {\overline{K}}_{\rho IV}, {\overline{K}}_{\rho V}$ are the matrix implementations of the five types of feasibility constraints identified above. Their calculation is most readily achieved through their associated 4th-order tensors.

**Type I Constraints:** For the Type I constraint, $\overline{{\mathcal {K}}}_{\rho I}$ is constructed from a sum of 4th-order outer products (Online Appendix Defn. 32) of elementary basis vectors.

39 $$\begin{aligned} \overline{{\mathcal {K}}}_{\rho I}&= \sum _{w_1=1}^{\sigma (P_\mu )} \sum _{v_1=1}^{\sigma (M)} \sum _{w_2=1} ^{\sigma (P_\mu )} \sum _{v_2=v_1}^{v_1} e_{w_1}^{\sigma (P)} \circ e_{v_1}^{\sigma (R)} \circ e_{w_2}^{\sigma (P)} \circ e_{v_2}^{\sigma (R)} \end{aligned}$$

where the $e_i^n$ notation places the value 1 on the *i*th element of a vector of length *n*. ${\overline{K}}_{\rho I}$ is calculated straightforwardly by matricizing both sides and evaluating the sums.

40 $$\begin{aligned} {\overline{K}}_{\rho I}&= \sum _{w_1=1}^{\sigma (P_\mu )} \sum _{v_1=1}^{\sigma (M)} \sum _{w_2=1} ^{\sigma (P_\mu )} \sum _{v_2=v_1}^{v_1} \left( e_{v_1}^{\sigma (R)} \otimes e_{w_1}^{\sigma (P)} \right) \otimes \left( e_{v_2}^{\sigma (R)} \otimes e_{w_2}^{\sigma (P)}\right) ^T \end{aligned}$$

41 $$\begin{aligned} {\overline{K}}_{\rho I}&= \sum _{v_1=1}^{\sigma (M)} \left( e_{v_1}^{\sigma (R)} \otimes \begin{array}{l} {{\mathbbm {1}}}^{\sigma (P_\mu )}\\ {{\mathbf {0}}}^{\sigma (P_{{\bar{\eta }}})} \end{array} \right) \left( e_{v_1}^{\sigma (R)} \otimes \begin{array}{l} {{\mathbbm {1}}}^{\sigma (P_\mu )}\\ {{\mathbbm {0}}}^{\sigma (P_{{\bar{\eta }}})} \end{array} \right) ^T \end{aligned}$$

**Type II Constraints:** Similarly, for the Type II constraint:

42 $$\begin{aligned} \overline{{\mathcal {K}}}_{\rho II}&= \sum _{w_1=1}^{\sigma (P_\mu )} \sum _{v_1=1}^{\sigma (M)} \sum _{y_1=v_1}^{v_1} \sum _{v_2=1}^{\sigma (R)} e_{w_1}^{\sigma (P)} \circ e_{v_1}^{\sigma (R)} \circ \begin{array}{l} {\mathbf {0}}^{\sigma (P_\mu )}\\ X_{y_1}^{\sigma (P_{\bar{\eta }})} \end{array} \circ e_{v_2}^{\sigma (R)} \end{aligned}$$

Here, the $X_{y_1}^{\sigma (P_{\bar{H}})}$ vector has a value of 1 wherever a refined transportation process $p_{w_2}$ originates at the transformation resource $m_{v_1}$. Drawing on the discussion of the 3rd-order tensor ${{\mathcal {J}}}_{\bar{H}}$ in “The system concept”, $X_{y_1}^{\sigma (P_{\bar{H}})}$, itself, is expressed as a vectorized sum of 3rd-order outer products.

43 $$\begin{aligned} X_{y_1}^{\sigma (P_{\bar{H}})}= \sum _{g=1}^{\sigma (P_\gamma )} \sum _{y_2=1}^{\sigma (B_S)} \left( e_g^{\sigma (P_\gamma )} \circ e_{y_1}^{\sigma (B_S)} \circ e_{y_2}^{\sigma (B_S)} \right) ^{TV}= \left( \mathbbm {1}^{\sigma (P_\gamma )} \otimes e_{y_1}^{\sigma (B_S)} \otimes \mathbbm {1}^{\sigma (B_S)} \right) \end{aligned}$$

${\overline{K}}_{\rho II}$ is then calculated straightforwardly by matricizing both sides of Eq. (42) and evaluating the sums.

44 $$\begin{aligned} {\overline{K}}_{\rho II}&= \sum _{w_1=1}^{\sigma (P_\mu )} \sum _{v_1=1}^{\sigma (M)} \sum _{y_1=v_1}^{v_1} \sum _{v_2=1}^{\sigma (R)} \left( e_{v_1}^{\sigma (R)} \otimes e_{w_1}^{\sigma (P)} \right) \otimes \left( e_{v_2}^{\sigma (R)} \otimes \begin{array}{l} {{\mathbf {0}}}^{\sigma (P_\mu )}\\ X_{y_1}^{\sigma (P_{\bar{H}})} \end{array} \right) ^T \end{aligned}$$

45 $$\begin{aligned} {\overline{K}}_{\rho II}&= \sum _{v_1=1}^{\sigma (M)} \left( e_{v_1}^{\sigma (R)} \otimes \begin{array}{ll} {{\mathbbm {1}}}^{\sigma (P_\mu )} \\ {{\mathbf {0}}}^{P_{\bar{\eta }}} \end{array} \right) \left( {\mathbbm {1}}^{\sigma (R)} \otimes \begin{array}{l} {{\mathbf {0}}}^{\sigma (P_\mu )}\\ {{\mathbbm {1}}}^{\sigma (P_\gamma )} \otimes e_{v_1}^{\sigma (B_S)} \otimes {{\mathbbm {1}}}^{\sigma (B_S)} \end{array} \right) ^T \end{aligned}$$

**Type III Constraints:** Similarly, for the Type III constraint:

46 $$\begin{aligned} \overline{{\mathcal {K}}}_{\rho III}&= \sum _{y_2=v_2}^{v_2} \sum _{v_1=1}^{\sigma (R)} \sum _{w_2=1}^{\sigma (P_\mu )} \sum _{v_2=1}^{\sigma (M)} \begin{array}{l} {\mathbf {0}}^{\sigma (P_\mu )}\\ X_{y_2}^{\sigma (P_{\bar{\eta }})} \end{array} \circ e_{v_1}^{\sigma (R)} \circ e_{w_2}^{\sigma (P)} \circ e_{v_2}^{\sigma (R)} \end{aligned}$$

Here, the $X_{y_2}^{\sigma (P_{\bar{H}})}$ vector has a value of 1 wherever a refined transportation process $p_{w_1}$ terminates at the transformation resource $m_{v_2}$. $X_{y_2}^{\sigma (P_{\bar{H}})}$, itself, is expressed as a vectorized sum of 3rd-order outer products.

47 $$\begin{aligned} X_{y_2}^{\sigma (P_{\bar{H}})}= \sum _{g=1}^{\sigma (P_\gamma )} \sum _{y_1=1}^{\sigma (B_S)} \left( e_g^{\sigma (P_\gamma )} \circ e_{y_1}^{\sigma (B_S)} \circ e_{y_2}^{\sigma (B_S)} \right) ^{TV}= \left( {\mathbbm {1}}^{\sigma (P_\gamma )} \otimes {\mathbbm {1}}^{\sigma (B_S)} \otimes e_{y_2}^{\sigma (B_S)} \right) \end{aligned}$$

${\overline{K}}_{\rho III}$ is then calculated straightforwardly by matricizing both sides of Eq. (46) and evaluating the sums.

48 $$\begin{aligned} \bar{K}_{\rho III}&= \sum _{y_2=v_2}^{v_2} \sum _{v_1=1}^{\sigma (R)} \sum _{w_2=1}^{\sigma (P_\mu )} \sum _{v_2=1}^{\sigma (M)} \left( e_{v_1}^{\sigma (R)} \otimes \begin{bmatrix} {\mathbf {0}}^{\sigma (P_\mu )}\\ X_{y_2}^{\sigma (P_{\bar{H}})} \end{bmatrix} \right) \otimes \left( e_{v_2}^{\sigma (R)} \otimes e_{w_2}^{\sigma (P)} \right) ^T \end{aligned}$$

49 $$\begin{aligned} {\overline{K}}_{\rho III}&= \sum _{v_2=1}^{\sigma (M)} \left( {{\mathbbm {1}}}^{\sigma (R)} \otimes \begin{bmatrix} {{\mathbf {0}}}^{\sigma (P_\mu )}\\ {{\mathbbm {1}}}^{\sigma (P_\gamma )} \otimes {{\mathbbm {1}}}^{\sigma (B_S)} \otimes e_{v_2}^{\sigma (B_S)} \end{bmatrix} \right) \left( e_{v_2}^{\sigma (R)} \otimes \begin{bmatrix} {{\mathbbm {1}}}^{\sigma (P_\mu )} \\ {{\mathbf {0}}}^{P_{\bar{\eta }}} \end{bmatrix} \right) ^T \end{aligned}$$

**Type IV Constraints:** Similarly, for the Type IV constraint:

50 $$\begin{aligned} \overline{{\mathcal {K}}}_{\rho IV}&= \sum _{y_2=1}^{\sigma {(B_S)}} \sum _{v_1=1}^{\sigma (R)} \sum _{y_1=y_2}^{y_2} \sum _{v_2=1}^{\sigma (R)} \begin{bmatrix} {\mathbf {0}}^{\sigma (P_\mu )}\\ X_{y_2}^{\sigma (P_{\bar{H}})} \end{bmatrix} \circ e_{v_1}^{\sigma (R)} \circ \begin{bmatrix} {\mathbf {0}}^{\sigma (P_\mu )}\\ X_{y_1}^{\sigma (P_{\bar{H}})} \end{bmatrix} \circ e_{v_2}^{\sigma (R)} \end{aligned}$$

${\overline{K}}_{\rho IV}$ is then calculated straightforwardly by matricizing both sides of Eq. (50) and evaluating the sums.

51 $$\begin{aligned} {\overline{K}}_{\rho IV}&= \sum _{y_2=1}^{\sigma {(B_S)}} \sum _{v_1=1}^{\sigma (R)} \sum _{y_1=y_2}^{y_2} \sum _{v_2=1}^{\sigma (R)} \left( e_{v_1}^{\sigma (R)} \otimes \begin{bmatrix} {{\mathbf {0}}}^{\sigma (P_\mu )}\\ X_{y_2}^{\sigma (P_{\bar{H}})} \end{bmatrix} \right) \otimes \left( e_{v_2}^{\sigma (R)} \otimes \begin{bmatrix} {{\mathbf {0}}}^{\sigma (P_\mu )}\\ X_{y_1}^{\sigma (P_{\bar{H}})} \end{bmatrix} \right) ^T \end{aligned}$$

52 $$\begin{aligned} {\overline{K}}_{\rho IV}&= \sum _{y_2=1}^{\sigma {(B_S)}} \left( {{\mathbbm {1}}}^{\sigma (R)} \otimes \begin{bmatrix} {{\mathbf {0}}}^{\sigma (P_\mu )}\\ {{\mathbbm {1}}}^{\sigma (P_\gamma )} \otimes {{\mathbbm {1}}}^{\sigma (B_S)} \otimes e_{y_2}^{\sigma (B_S)} \end{bmatrix} \right) \left( {{\mathbbm {1}}}^{\sigma (R)} \otimes \begin{bmatrix} {{\mathbf {0}}}^{\sigma (P_\mu )}\\ {{\mathbbm {1}}}^{\sigma (P_\gamma )} \otimes e_{y_2}^{\sigma (B_S)} \otimes {{\mathbbm {1}}}^{\sigma (B_S)} \end{bmatrix} \right) ^T \end{aligned}$$

**Type V Constraints:** The Type V constraint must make use of the functional graph adjacency matrix $A_P$. Consequently, the fourth-order tensor ${{\mathcal {K}}}_{\rho V}$ is calculated first on a scalar basis using the Kronecker delta function $\delta _i$ (Online Appendix Defn. 30) and then is matricized to $K_{\rho V}$.

53 $$\begin{aligned} {{\overline{{\mathcal {K}}}}}_{\rho V}(w_1,v_1,w_2,v_2)&= \delta _{v_1v_1} \cdot \delta _{v_2v_2} \cdot A_P(w_1,w_2) \qquad \forall w_1,w_2 \in \{1, \ldots , \sigma (P)\}, \; v_1,v_2 \in \{1, \ldots , \sigma (R)\} \end{aligned}$$

54 $$\begin{aligned} {\overline{K}}_{\rho V}&= \sum _{v_1=1}^{\sigma (R)} \sum _{v_2=1}^{\sigma (R)} \left( e_{v_1}^{\sigma (R)} \otimes e_{v_2}^{\sigma (R)T} \right) \otimes A_P \end{aligned}$$

55 $$\begin{aligned} {\overline{K}}_{\rho V}&= \left( {\mathbbm {1}}^{\sigma (R)} \otimes {\mathbbm {1}}^{\sigma (R)T} \right) \otimes A_P \end{aligned}$$

### Sequence-dependent degrees of freedom

Once the system sequence knowledge base and constraints matrix have been calculated, the number of sequence-dependent degrees of freedom follow straightforwardly.

#### Definition 12

(*Sequence-Dependent Degrees of Freedom*^13,108,117–119^) The set of independent pairs of actions $z_{\chi _1\chi _2}=e_{w_1v_1}e_{w_2v_2} \in {{\mathcal {Z}}}$ of length 2 that completely describe the system language. The number is given by:

56 $$\begin{aligned} DOF_{\rho } = \sigma ({{\mathcal {Z}}})&= \sum _{\chi _1}^{\sigma (R)\sigma (P)} \sum _{\chi _2}^{\sigma (R)\sigma (P)} [J_\rho \ominus K_\rho ](\chi _1,\chi _2) \end{aligned}$$

57 $$\begin{aligned}&=\sum _{\chi _1}^{\sigma (R)\sigma (P)}\sum _{\chi _2}^{\sigma (R)\sigma (P)}[A_\rho ](\chi _1,\chi _2) \end{aligned}$$

For systems of substantial size, the size of the hetero-functional adjacency matrix may be challenging to process computationally. However, the matrix is generally very sparse. Therefore, projection operators are used to eliminate the sparsity by projecting the matrix onto a one’s vector^108,119^. This is demonstrated below for $J_S^V$ and $A_\rho$:

58 $$\begin{aligned} \mathbb {P}_S J_S^V&= \mathbbm {1}^{\sigma ({{\mathcal {E}}}_S)} \end{aligned}$$

59 $$\begin{aligned} \mathbb {P}_S A_\rho \mathbb {P}_S^T&= {\widetilde{A}}_{\rho } \end{aligned}$$

where $\mathbb {P}_S$ is a (non-unique) projection matrix for the vectorized system knowledge base and the hetero-functional adjacency matrix^108,119^. Note that the number of sequence dependent degrees of freedom for the projected hetero-functional adjacency matrix can be calculated as:

60 $$\begin{aligned} DOF_{\rho } = \sigma ({{\mathcal {Z}}}) = \sum _{\psi _1}^{\sigma ({{\mathcal {E}}}_S)} \sum _{\psi _2}^{\sigma ({{\mathcal {E}}}_S)} [{\widetilde{A}}_\rho ](\psi _1,\psi _2) \end{aligned}$$

where $$\psi \in \left[ 1, \dots ,\sigma ({{\mathcal {E}}}_S)\right]$$.

## Hetero-functional incidence tensor

This section serves to introduce the hetero-functional incidence tensor as part of the paper’s original contribution. “Third order form” describes the tensor in third-order form. “Fourth order form” then elaborates why it sometimes useful to present this tensor in fourth-order form. Finally, “Second order form” shows how matricizing the heter-functional incidence tensor (into second-order form) can serve to reconstruct the hetero-functional adjacency matrix.

### Third order form

To complement the concept of a hetero-functional adjacency matrix $A_\rho$ and its associated tensor ${{\mathcal {A}}}_\rho$, the hetero-functional incidence tensor $\widetilde{{\mathcal {M}}}_\rho$ describes the structural relationships between the physical capabilities (i.e. structural degrees of freedom) ${{\mathcal {E}}}_S$, the system operands *L*, and the system buffers $B_S$.

61 $$\begin{aligned} \widetilde{{\mathcal {M}}}_\rho =\widetilde{{\mathcal {M}}}_\rho ^+-\widetilde{{\mathcal {M}}}_\rho ^- \end{aligned}$$

#### Definition 13

(*The Negative* 3rd *Order Hetero-functional Incidence Tensor* $\widetilde{{\mathcal {M}}}_\rho ^-$) The negative hetero-functional incidence tensor $\widetilde{{\mathcal {M}}_\rho }^- \in \{0,1\}^{\sigma (L)\times \sigma (B_S) \times \sigma ({{\mathcal {E}}}_S)}$ is a third-order tensor whose element $\widetilde{{\mathcal {M}}}_\rho ^{-}(i,y,\psi )=1$ when the system capability ${\epsilon }_\psi \in {{\mathcal {E}}}_S$ pulls operand $l_i \in L$ from buffer $b_{s_y} \in B_S$.

#### Definition 14

(*The Positive* 3rd *Order Hetero-functional Incidence Tensor* $\widetilde{{\mathcal {M}}}_\rho ^+$) The positive hetero-functional incidence tensor ${\widetilde{{\mathcal {M}}}}_\rho ^+ \in \{0,1\}^{\sigma (L)\times \sigma (B_S) \times \sigma ({{\mathcal {E}}}_S)}$ is a third-order tensor whose element $\widetilde{{\mathcal {M}}}_\rho ^{+}(i,y,\psi )=1$ when the system capability ${\epsilon }_\psi \in {{\mathcal {E}}}_S$ injects operand $l_i \in L$ into buffer $b_{s_y} \in B_S$.

The calculation of these two tensors depends on the definition of two more matrices which further depend on the hetero-functional graph theory definitions in Appendix C.

#### Definition 15

(*The Negative Process-Operand Incidence Matrix* $M_{LP}^-$) A binary incidence matrix $M_{L P}^{-} \in \{0,1\}^{\sigma (L)\times \sigma (P)}$ whose element $M_{L P}^{-}(i,w)=1$ when the system process $p_w \in P$ pulls operand $l_i \in L$ as an input. It is further decomposed into the negative transformation process-operand incidence matrix $M_{L P_\mu }^-$ (Definition 14) and the negative refined transformation process-operand incidence matrix $M_{L P_{\bar{\eta }}}^-$ (Definition 15) which by definition is in turn calculated from the negative holding process-operand incidence matrix $M_{LP_{\gamma }}^-$ (Definition 16).

62 $$\begin{aligned} M_{L P}^-= \begin{bmatrix} M_{L P_\mu }^{-}&M_{L P_{\bar{\eta }}}^- \end{bmatrix} = \begin{bmatrix} M_{L P_\mu }^{-}&M_{LP_{\gamma }}^- \otimes \mathbbm {1}^{\sigma (P_\eta )T} \end{bmatrix} \end{aligned}$$

#### Definition 16

(*The Positive Process-Operand Incidence Matrix* $M_{LP}^+$) A binary incidence matrix $M_{LP}^{+} \in \{0,1\}^{\sigma (L)\times \sigma (P)}$ whose element $M_{L P}^{+}(i,w)=1$ when the system process $p_w \in P$ injects operand $l_i \in L$ as an output. It is further decomposed into the positive transformation process-operand incidence matrix $M_{LP_\mu }^+$ (Definition 17) and the positive refined transformation process-operand incidence matrix $M_{LP_{\bar{\eta }}}^+$ (Definition 18) which, by definition, is, in turn, calculated from the positive holding process-operand incidence matrix $M_{LP_{\gamma }}^+$ (Definition 19)

63 $$\begin{aligned} M_{LP}^+= \begin{bmatrix} M_{LP_\mu }^{+}&M_{LP_{\bar{\eta }}}^+ \end{bmatrix} = \begin{bmatrix} M_{LP_\mu }^{+}&M_{LP_{\gamma }}^+ \otimes \mathbbm {1}^{\sigma (P_\eta )T} \end{bmatrix} \end{aligned}$$

With the definitions of these incidence matrices in place, the calculation of the negative and positive hetero-functional incidence tensors $\widetilde{{\mathcal {M}}}_\rho ^-$ and $\widetilde{{\mathcal {M}}}_\rho ^+$ follows straightforwardly as a third-order outer product. For $\widetilde{{\mathcal {M}}}_\rho ^-$:

64 $$\begin{aligned} {\widetilde{{\mathcal {M}}}}_\rho ^-=\sum _{i=1}^{\sigma (L)}\sum _{y_1=1}^{\sigma (B_S)} e_i^{\sigma (L)} \circ e_{y_1}^{\sigma (B_S)} \circ \mathbb {P}_S \left( \left( X^{-}_{i y_1} \right) ^V\right) \end{aligned}$$

where

65 $$\begin{aligned} X^{-}_{i y_1}= \left[ \begin{array}{l} M_{LP_\mu }^{-T}e_{i}^{\sigma (L)}e_{y_1}^{\sigma (M)T} \quad | \quad {\mathbf {0}}\\ \hline M_{LP_{\gamma }}^{-T}e_{i}^{\sigma (L)} \otimes \left( e_{y_1}^{\sigma (B_S)} \otimes \mathbbm {1}^{\sigma (B_S)}\right) \otimes \mathbbm {1}^{\sigma (R)T}\\ \end{array}\right] \end{aligned}$$

The $X^{-}_{i y_1}$ matrix is equivalent in size to the system concept $A_S$. It has a value of one in all elements where the associated process both withdraws input operand $l_i$ and originates at the buffer $b_{s_{y_1}}$. Consequently, when $X^{-}_{i y_1}$ is vectorized and then projected with $\mathbb {P}_S$, the result is a vector with a value of one only where the associated system capabilities meet these criteria.

For $$\widetilde{{\mathcal {M}}}_\rho ^+$$:

66 $$\begin{aligned} {\widetilde{{\mathcal {M}}}}_\rho ^+=\sum _{i=1}^{\sigma (L)}\sum _{y_2=1}^{\sigma (B_S)} e_i^{\sigma (L)} \circ e_{y_2}^{\sigma (B_S)} \circ \mathbb {P}_S \left( \left( X^{+}_{i y_2} \right) ^V\right) \end{aligned}$$

where

67 $$\begin{aligned} X^{+}_{i y_2}= \left[ \begin{array}{l} M_{LP_\mu }^{+T}e_{i}^{\sigma (L)}e_{y_2}^{\sigma (M)T} \quad | \quad {\mathbf {0}}\\ \hline M_{LP_{\gamma }}^{+T}e_{i}^{\sigma (L)} \otimes \left( \mathbbm {1}^{\sigma (B_S)} \otimes e_{y_2}^{\sigma (B_S)}\right) \otimes \mathbbm {1}^{\sigma (R)T}\\ \end{array}\right] \end{aligned}$$

The $X^{+}_{i y_2}$ matrix is equivalent in size to the system concept $A_S$. It also has a value of one in all elements where the associated process both injects output operand $l_i$ and terminates at the buffer $b_{s_{y_2}}$. Consequently, when $X^{+}_{i y_2}$ is vectorized and then projected with $\mathbb {P}_S$, the result is a vector with a value of one only where the associated system capabilities meet these criteria.

It is important to note that the definitions of the 3rd order hetero-functional incidence tensors $\widetilde{{\mathcal {M}}}_\rho ^-$, and $\widetilde{{\mathcal {M}}}_\rho ^+$ are provided in projected form as indicated by the presence of the projection operator $\mathbb {P}_S$ in Eqs. (64) and (66) respectively. It is often useful to use the un-projected form of these tensors.

68 $$\begin{aligned} {{\mathcal {M}}}_\rho ^-&=\sum _{i=1}^{\sigma (L)}\sum _{y_1=1}^{\sigma (B_S)} e_i^{\sigma (L)} \circ e_{y_1}^{\sigma (B_S)} \circ \left( X^{-}_{i y_1} \right) ^V \end{aligned}$$

69 $$\begin{aligned} {{\mathcal {M}}}_\rho ^+&=\sum _{i=1}^{\sigma (L)}\sum _{y_2=1}^{\sigma (B_S)} e_i^{\sigma (L)} \circ e_{y_2}^{\sigma (B_S)} \circ \left( X^{+}_{i y_2} \right) ^V \end{aligned}$$

### Fourth order form

The third dimension of these unprojected 3rd order hetero-functional incidence tensors can then be split into two dimensions to create 4th order hetero-functional incidence tensors.

70 $$\begin{aligned} {{\mathcal {M}}}_{PR}^+&=vec^{-1}\left( {{\mathcal {M}}}_\rho ^+,[\sigma (P),\sigma (R)],3\right) \end{aligned}$$

71 $$\begin{aligned} {{\mathcal {M}}}_{PR}^-&=vec^{-1}\left( {{\mathcal {M}}}_\rho ^-,[\sigma (P),\sigma (R)],3\right) \end{aligned}$$

These fourth order tensors describe the structural relationships between the system processes *P*, the physical resources *R* that realize them, the system operands *L* that are consumed and injected in the process, and the system buffers $B_S$ from which these are operands are sent and the system buffers $B_S$ to which these operands are received. They are used in the following section as part of the discussion on layers.

72 $$\begin{aligned} {{\mathcal {M}}}_{PR}={{\mathcal {M}}}_{PR}^+ - {{\mathcal {M}}}_{PR}^- \end{aligned}$$

#### Definition 17

(*The Negative* 4th *Order Hetero-functional Incidence Tensor* ${{\mathcal {M}}}_{PR}^-$) The negative 4th Order hetero-functional incidence tensor ${{\mathcal {M}}}_{PR}^- \in \{0,1\}^{\sigma (L)\times \sigma (B_S) \times \sigma (P) \times \sigma (R)}$ has element ${{\mathcal {M}}}_{PR}^{-}(i,y,w,v)=1$ when the system process $p_w \in P$ realized by resource $r_v \in R$ pulls operand $l_i \in L$ from buffer $b_{s_y} \in B_S$.

#### Definition 18

(*The Positive* 4th *Order Hetero-functional Incidence Tensor* ${{\mathcal {M}}}_{PR}^-$) The positive 4th Order hetero-functional incidence tensor ${{\mathcal {M}}}_{PR}^- \in \{0,1\}^{\sigma (L)\times \sigma (B_S) \times \sigma (P) \times \sigma (R)}$ has element ${{\mathcal {M}}}_{PR}^{-}(i,y,w,v)=1$ when the system process $p_w \in P$ realized by resource $r_v \in R$ injects operand $l_i \in L$ into buffer $b_{s_y} \in B_S$.

Furthermore, the negative and positive 4th hetero-functional incidence tensors can be used to demonstrate a direct relationship to the system concept $A_S$.

73 $$\begin{aligned} {{\mathcal {M}}}_{PR}^{-}(i,y,w,v) =&X^{-}_{i y_1}(w,v) \cdot A_S(w,v) \end{aligned}$$

74 $$\begin{aligned} {{\mathcal {M}}}_{PR}^{-} =&\sum _i^{\sigma (L)}\sum _{y_1}^{\sigma (B_S)} X^{-}_{i y_1} \cdot A_S \end{aligned}$$

75 $$\begin{aligned} {{\mathcal {M}}}_{PR}^{+}(i,y,w,v) =&X^{+}_{i y_2}(w,v) \cdot A_S(w,v) \end{aligned}$$

76 $$\begin{aligned} {{\mathcal {M}}}_{PR}^{+} =&\sum _i^{\sigma (L)}\sum _{y_2}^{\sigma (B_S)} X^{+}_{i y_2} \cdot A_S \end{aligned}$$

Equations (74) and (76) show that the 4th order hetero-functional incidence tensors contain three types of information:

1. the mapping of system processes to system resources in the system concept $A_S$,
2. the mapping of processes to their operands in $M_{LP}^-$ and $M_{LP}^+$,
3. the implicit knowledge that by definition transformation processes occur at a stationary buffer, and that transportation processes are defined by their origin and destination buffers.

In other words, the hetero-functional incidence tensor is a complete descption of a system’s allocated architecture.

### Second order form

Returning back to the third-order hetero-functional incidence tensor ${\widetilde{M}}_\rho$, it and and its positive and negative components ${\widetilde{M}}_\rho ^+, {\widetilde{M}}_\rho ^-$, can also be easily matricized.

77 $$\begin{aligned} M_\rho&={{\mathcal {F}}}_M\left( {{\mathcal {M}}}_\rho ,[1,2],[3]\right) \end{aligned}$$

78 $$\begin{aligned} M_\rho ^-&={{\mathcal {F}}}_M\left( {{\mathcal {M}}}_\rho ^-,[1,2],[3]\right) \end{aligned}$$

79 $$\begin{aligned} M_\rho ^+&={{\mathcal {F}}}_M\left( {{\mathcal {M}}}_\rho ^+,[1,2],[3]\right) \end{aligned}$$

The resulting matrices have a size of $\sigma (L)\sigma (B_S) \times \sigma (\mathcal {E_S})$ which have a corresponding physical intuition. Each buffer $b_{s_{y}}$ has $\sigma (L)$ copies to reflect a place (i.e. bin) for each operand at that buffer. Each of these places then forms a bipartite graph with the system’s physical capabilities. Consequently, and as expected, the hetero-functional adajacency matrix $A_\rho$ can be calculated as a matrix product of the positive and negative hetero-functional incidence matrices $M_\rho ^+$ and $M_\rho ^+$.

80 $$\begin{aligned} A_\rho = M_\rho ^{+T}\odot M_\rho ^- = M_\rho ^{+T}M_\rho ^- \end{aligned}$$

Such a product systematically enforces all five of the feasibility constraints identified in “Hetero-functional adjacency matrix”. Furthermore, the Boolean and real matrix products are interchangeable because each process is associated with exactly one origin-destination pair.

## Discussion

Given the discussion on multi-layer networks in the introduction, it is worthwhile reconciling the gap in terminology between multi-layer networks and hetero-functional graph theory. First, the concept of layers in hetero-functional graphs is discussed. Second, an ontological comparison of layers in hetero-functional graphs and multi-layer networks is provided. Third, a discussion of network descriptors in the context of layers is provided. Given the “disparate terminology and the lack of consensus” in the multi-layer network literature, the discussion uses the multi-layer description provided De Dominico et. al^14^.

### Layers in hetero-functional graphs

#### Definition 19

(*Layer*^120^) A layer $G_{\lambda } = \{{{\mathcal {E}}}_{S\lambda }, Z_{S\lambda } \}$ of a hetero-functional graph $G = \{{{\mathcal {E}}}_{S}, Z_{S}\}$ is a subset of a hetero-functional graph, $G_{\lambda } \subseteq G$, for which a predefined layer selection (or classification) criterion applies. A set of layers in a hetero-functional graph adhere to a classification scheme composed of a number of selection criteria.

Note that this definition of a layer is particularly flexible because it depends on the nature of the classification scheme and its associated selection criteria. Nevertheless, and as discussed later, it is important to choose a classification scheme that leads to a set of mutually exclusive layers that are also collectively exhaustive of the hetero-functional graph as a whole.

To select out specific subsets of capabilities (or structural degrees of freedom), HFGT has used the concept of “selector matrices” of various types^7,121^. Here a layer selector matrix is defined.

#### Definition 20

^120^ Layer Selector Matrix: A binary matrix $\Lambda _\lambda$ of size $\sigma (P)\times \sigma (R)$ whose element $\Lambda _\lambda (w,v)=1$ when the capability $e_{wv} \subset E_{S\lambda }$.

From this definition, the calculation of a hetero-functional graph layer follows straightforwardly. First, a layer projection operator $\mathbb {P}_\lambda$ is calculated^120^:

81 $$\begin{aligned} \mathbb {P}_\lambda \Lambda _\lambda ^V&= \mathbbm {1}^{\sigma ({{\mathcal {E}}}_S)} \end{aligned}$$

Next, the negative and positive hetero-functional incidence tensors $\widetilde{{\mathcal {M}}}_{\rho \lambda }^-$ and ${\widetilde{{\mathcal {M}}}}_{\rho \lambda }^+$ for a given layer $\lambda$ are calculated straightforwardly^120^.

82 $$\begin{aligned} \widetilde{{\mathcal {M}}}_{\rho \lambda }^-&=\sum _{i=1}^{\sigma (L)}\sum _{y_1=1}^{\sigma (B_S)} e_i^{\sigma (L)} \circ e_{y_1}^{\sigma (B_S)} \circ \mathbb {P}_\lambda \left( \left( X^{-}_{i y_1} \right) ^V\right) = {\widetilde{{\mathcal {M}}}}_{\rho }^- \odot _3 \mathbb {P}_\lambda \end{aligned}$$

83 $$\begin{aligned} \widetilde{{\mathcal {M}}}_{\rho \lambda }^+&=\sum _{i=1}^{\sigma (L)}\sum _{y_2=1}^{\sigma (B_S)} e_i^{\sigma (L)} \circ e_{y_2}^{\sigma (B_S)} \circ \mathbb {P}_S \left( \left( X^{+}_{i y_2} \right) ^V\right) = {\widetilde{{\mathcal {M}}}}_{\rho }^+ \odot _3 \mathbb {P}_\lambda \end{aligned}$$

From there, the positive and negative hetero-functional incidence tensors for a given layer can be matricized and the adjacency matrix of the associated layer ${\widetilde{A}}_{\rho \lambda }$ follows straightforwardly^120^.

84 $$\begin{aligned} {\widetilde{M}}_{\rho \lambda }^+&= {{\mathcal {F}}}_M (\widetilde{{\mathcal {M}}}_{\rho \lambda }^+, [1,2],[3]) \end{aligned}$$

85 $$\begin{aligned} {\widetilde{M}}_{\rho \lambda }^-&= {{\mathcal {F}}}_M (\widetilde{{\mathcal {M}}}_{\rho \lambda }^-, [1,2],[3]) \end{aligned}$$

86 $$\begin{aligned} {\widetilde{A}}_{\rho \lambda }&= {\widetilde{M}}_{\rho \lambda }^{+ T} \odot {\widetilde{M}}_{\rho \lambda }^{-} \end{aligned}$$

This approach of separating a hetero-functional graph into its constituent layers is quite generic because the layer selector matrix $\Lambda _\lambda$ can admit a wide variety of classification schemes. Three classification schemes are discussed here:

1. An Input Operand Set Layer
2. An Output Operand Set Layer
3. A Dynamic Device Model Layer

**Figure 4:**
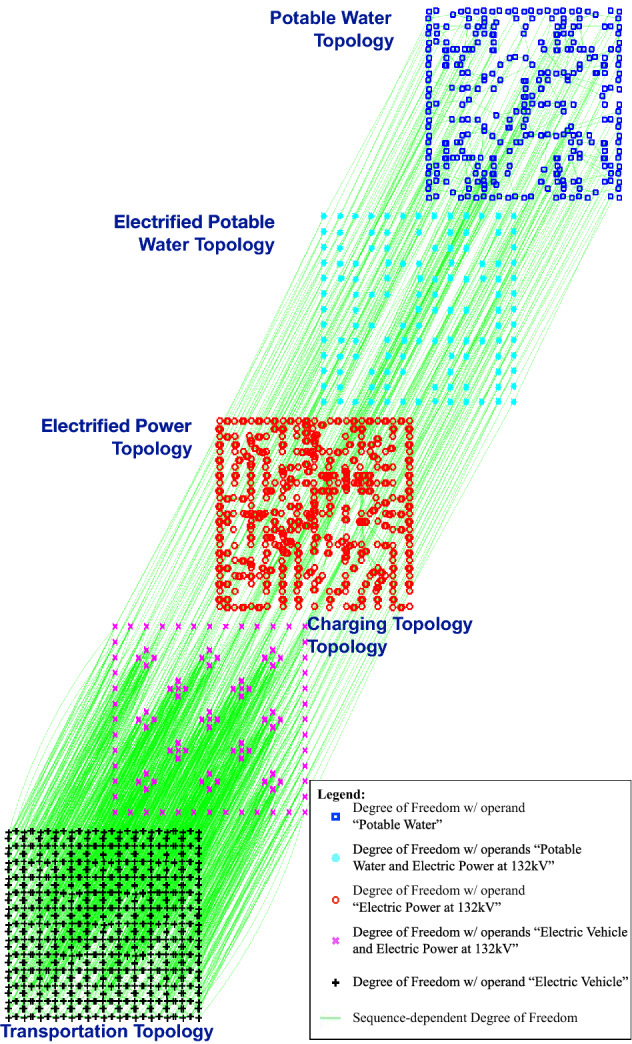
The trimetric smart city infrastructure test case visualized as five layers defined by input operand sets: the potable water topology, the electrified potable water topology, the electric power topology, the charging topology, and the transportation topology.

#### Definition 21

Input Operand Set Layer: A hetero-functional graph layer for which all of the node-capabilities have a common set of input operands $L_\lambda \subseteq L$.

This definition of an Operand Set Layer was used in the HFGT text^7^ to partition the Trimetrica test case (first mentioned in Fig. 1) into the multi-layer depiction in Fig. 4. In this classification scheme, any system contains up to 2$^{\sigma (L)}$ possible layers. For completeness, an index $\lambda _D \in \{1, \dots , 2^{\sigma (L)}\}$ is used to denote a given layer. In reality, however, the vast majority of physical systems exhibit far fewer than 2$^{\sigma (L)}$ layers. Consequently, it is often useful to simply assign an index $\lambda$ to each layer and create a 1-1 mapping function (i.e. lookup table) $f_\lambda$ back to the $\lambda _D$ index.

87 $$\begin{aligned} f_\lambda{:} \lambda \rightarrow \lambda _D \end{aligned}$$

The utility of the $\lambda _d$ index (stated as a base 10 number) becomes apparent when it is converted into a binary (base 2) number $\lambda _v \in \{0,1\}^{\sigma (L)}$ which may be used equivalently as a binary vector of the same length.

88 $$\begin{aligned} \lambda _v = bin(\lambda _D) \end{aligned}$$

The resulting binary vector $\lambda _v$ has the useful property that $\lambda _v (l_i) = 1$, iff operand $l_i \in L_\lambda$. Consequently, a given value of $\lambda _v$ serves to select from *L* the operands that pertain to layer $\lambda$. The associated layer selector matrix follows straightforwardly:

89 $$\begin{aligned} \Lambda _\lambda (w,v)= \left\{ \begin{array}{cc} 1 &{} \text{ if } \quad \lambda _v = M_{LP}^-(:,w) \quad \forall r_v \in R\\ 0 &{} \text{ otherwise } \; \end{array} \right. \end{aligned}$$

It is also worth noting that the layer selector matrix $\Lambda$ above is effectively a third order tensor whose value $\Lambda (\lambda ,w,v)=1$ when the capability $e_{wv}$ is part of layer $\lambda$.

One advantage of a classification scheme based on ***sets*** of input operands is that they lead to the generation of a mutually exclusive and collectively exhaustive set of layers. Because no process (and consequently capability) has two sets of input operands, it can only exist in a single layer (mutual exclusivity). In the meantime, the presence of $2^{\sigma (L)}$ assures that all capabilities fall into (exactly) one layer (exhaustivity). It is worth noting that a classification scheme based on ***individual*** operands would not yield these properties. For example, a water pump consumes electricity and water as input operands. Consequently, it would have a problematic existence in both the “water layer” as well as the “electricity layer”. In contrast, a classification scheme based on operand sets creates an “electricity-water” layer.

Analogously to Definition 21, an output-operand set layer can be defined and its associated layer selector matrix calculated.

#### Definition 22

Output Operand Set Layer: A hetero-functional graph layer for which all of the node-capabilities have a common set of output operands $L_\lambda \subseteq L$.

90 $$\begin{aligned} \Lambda _\lambda (w,v)= \left\{ \begin{array}{ll} 1 &{} \text{ if } \quad \lambda _v = M_{LP}^+(:,w) \quad \forall r_v \in R\\ 0 &{}\quad \text{ otherwise } \; \end{array} \right. \end{aligned}$$

The third classification scheme is required when developing dynamic equations of motion from the structural information of a hetero-functional graph^117,122^. Every process is said to have a “dynamic device model” that is usually described as a set of differential, algebraic, or differential-algebraic equations^117,122^. The simplest of these are the constitutive laws of basic dynamic system elements (e.g. resistors, capacitors, and inductors). Some processes, although distinct, may have device models with the same functional form. For example, two resistors at different places in an electrical system have the same constitutive (Ohm’s) law, but have different transportation processes because their origin and destinations are different. Consequently, layers that distinguish on the basis of dynamic device model (i.e. constitutive law) are necessary.

#### Definition 23

Dynamic Device Model Layer: A hetero-functional graph layer for which all of the node-capabilities have a dynamic device model with the same functional form.

In such a case, the layer selector matrix $\Lambda _\lambda$ straightforwardly maps capabilities to their layer and dynamic device model interchangeably. A sufficient number of layers need to be created to account for all of the different types of dynamic device models in the system. This classification scheme may be viewed as a generalization of the well-known literature on “linear-graphs”^123^ and “bond graphs”^124^.

### Finding commonality between multilayer networks and hetero-functional graphs

The above discussion of layers in a hetero-functional graph inspires a comparison with multi-layer networks. The multi-layer adjacency tensor (${{\mathcal {A}}}_{MLN}$) defined by De Dominico et. al.^14^ is chosen to facilitate the discussion. This fourth order tensor has elements ${{\mathcal {A}}}_{MLN}(\alpha _1,\alpha _2,\beta _1,\beta _2)$ where the indices $\alpha _1,\alpha _2$ denote “vertices” and $\beta _1,\beta _2$ denote “layers”. De Dominico et. al write that this multilayer adjacency tensor is a^14^: *“...very general object that can be used to represent a wealth of complicated relationships among nodes.”* The challenge in reconciling the multi-layer adjacency tensor ${{\mathcal {A}}}_{MLN}$ and the hetero-functional adjacency tensor ${{\mathcal {A}}}_{\rho }$ is an ontological one. Referring back to the ontological discussion in the introduction and more specifically Fig. 2 reveals that the underlying abstract conceptual elements (in the mind) to which these two mathematical models refer may not be the same.

Consider the following interpretation of ${{\mathcal {A}}}_{MLN}(\alpha _1,\alpha _2,\beta _1,\beta _2)= {{\mathcal {A}}}_{B_Sl_1}(y_1,y_2,i_1,i_2)$ where the multi-layer network’s vertices are equated to the buffers $B_S$ and the layers are equated to the operands *L*. This interpretation would well describe the departure of an operand $l_{i_1}$ from buffer $b_{sy_1}$ and arriving as $l_{i_2}$ at $b_{sy_2}$. The equivalence of vertices to buffers is effectively a consensus view in the literature. In contrast, the concept of a “layer” in a multi-layer network (as motivated in the introduction) remains relatively unclear. The equivalence of layers to operands warrants further attention.

#### Theorem 1

The mathematical model $A_{B_Sl_1}$ is neither lucid nor complete with respect to the system processes *P* (as an abstraction).

#### Proof

By contradiction. Assume that ${{\mathcal {A}}}_{B_Sl_1}$ is both lucid and complete network model with respect to system processes *P*. Consider an operand $l_1$ that departs $b_{s1}$, undergoes process $p_1$, and arrives as $l_1$ at $b_{s2}$. Now consider the same operand $l_1$ that departs $b_{s1}$, undergoes process $p_2$, and arrives as $l_1$ at $b_{s2}$. Both of these scenarios would be denoted by ${{\mathcal {A}}}_{B_Sl_1}(1,2,1,1)=1$. Consequently, this modeling element is overloaded and as such violates the ontological property of lucidity. Furthermore, because ${{\mathcal {A}}}_{B_Sl_1}$ makes no mention of the concept of system processes, then it violates the completeness property as well. $\square$

**Figure 5:**
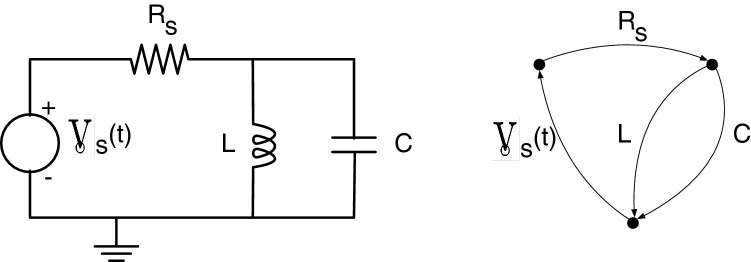
A simple RLC circuit shown as a circuit diagram on left and as a linear graph model on right. Each resistor, capacitor and inductor can be said to be part of its own layer by virtue of their distinct constitutive laws.

The counter-example provided in the proof above is not simply a theoretical abstraction but rather quite practical. For several decades, the field of mechanical engineering has used “linear graphs”^123^ to derive the equations of motion of dynamic systems with multi-domain physics. Consider the RLC circuit shown in Fig. 5 and its associated linear graph. As parallel elements, the inductor and capacitor both transfer electrical power (as an operand) between the same pair of nodes. However, the constitutive law (as a physical process) of a capacitor is distinct from that of the inductor. Consequently, the interpretation ${{\mathcal {A}}}_{B_Sl_1}$ of a multi-layer network is inadequate even for this very simple counter-example (Although electric power systems and circuits have served as a rich application domain for graph theory and network science, these approaches usually parameterize the circuit components homogeneously as a fixed-value impedance/admittance at constant frequency. When the constant frequency assumption is relaxed, the diversity of constitutive laws for resistors, capacitors, and inductors must be explicitly considered.).

Another possible interpretation of a multi-layer network is ${\mathcal {A}}_{MLN}(\alpha _1,\alpha _2,\beta _1,\beta _2)$ = ${\mathcal {A}}_{B_Sl_2}(y_1,y_2,w_1,w_2)$ where the multi-layer network’s vertices are equated to the buffers $B_S$ and the layers are equated to the processes *P*. This interpretation would well describe the execution of a process $p_{w_1}$ that is realized by buffer $b_{sy_1}$ followed by a process $p_{w_2}$ that is realized by buffer $b_{sy_2}$. The equivalence of layers to processes warrants further attention as well.

#### Theorem 2

The mathematical model $A_{B_Sl_2}$ is neither lucid nor complete with respect to the system’s transportation resources *H* (as an abstraction).

#### Proof

By contradiction. Assume that ${{\mathcal {A}}}_{B_Sl_2}$ is both a lucid and complete network model with respect to system’s transportation resources *H*. Consider transportation process *p* between a buffer $b_{s1}$ and a distinct buffer $b_{s2}$. If such a transportation process were realized by any buffer $b_s \in B_S$, then by definition it would no longer be a buffer but rather a transportation resource. Consequently, ${{\mathcal {A}}}_{B_Sl_2}$ is not complete with respect the system’s transportation resources *H*. Now consider a process $p_{1}$ that is realized by buffer $b_{s1}$ followed by a process $p_{2}$ that is realized by a distinct buffer $b_{s2}$. This is denoted by ${{\mathcal {A}}}_{B_Sl_2}(1,2,1,2)=1$. Given the distinctness of $b_{s1}$ and $b_{s2}$, a transportation process must have happened in between $p_1$ and $p_2$ although it is not explicitly stated by the mathematical statement ${\mathcal {A}}_{B_Sl_2}(1,2,1,2)=1$. Such a transportation process, although well-defined by its origin and destination could have been realized by any one of a number of transportation resources. Consequently, the modeling element is overloaded and as such violates the property of lucidity. The lack of an explicit description of transportation processes or resources limits the utility of this type of multi-layer network model. $\square$

It is worth noting that the first multi-layer network interpretation ${{\mathcal {A}}}_{B_Sl_1}$ can be derived directly from the positive and negative hetero-functional incidence matrices^120^.

91 $$\begin{aligned} {{\mathcal {A}}}_{B_SL_1}(y_1,y_2,i_1,i_2)&= \bigvee _\psi {{\mathcal {M}}}_\rho ^-(i,y_1,\psi )\cdot {{\mathcal {M}}}_\rho ^+(i,y_2,\psi ) \end{aligned}$$

92 $$\begin{aligned} {{\mathcal {A}}}_{B_Sl_1}&= {{\mathcal {F}}}_M^{-1}\left( M_\rho ^{-T}\odot M_\rho ^+, [\sigma (B_S),\sigma (B_S),\sigma (L),\sigma (L)],[1,3],[2,4]\right) \end{aligned}$$

When $M_\rho ^{-T}$ and $M_\rho ^+$ are multiplied so that the capabilities ${{\mathcal {E}}}_s$ are the inner dimension, the result is an adjacency matrix that when tensorized becomes ${{\mathcal {A}}}_{B_Sl_1}$. In effect, ${A}_{B_Sl_1}$ (in matricized form) is the dual adjacency matrix^125^ of the hetero-functional adjacency matrix $A_\rho$. The presence of this matrix multiplication obfuscates (i.e. creates a lack of lucidity) as to whether one capability or another occurred when expressing the adjacency tensor element ${{\mathcal {A}}}_{B_Sl_1}(y_1,y_2,i_1,i_2)$. In contrast, the matrix multiplication in Eq. (80) does not cause the same problem. When two capabilities succeed one another, the information associated with their physical feasibility in terms of intermediate buffers and their functional feasibility in terms of intermediate operands remains intact. In other words, given the sequence of capabilities $e_{w_1v_1}e_{w_2v_2}$, one can immediately deduce the exchanged operands in $L_\lambda \subseteq L$ and the intermediate buffer $b_s$. In the case of the exchanged operands, one simply needs to intersect the output-operand set of the first process with the input operand set of the second process. In the case of the intermediate buffer, one checks if either or both of the resources are buffers. If not, then two transportation processes followed one another and the intermediate buffer is deduced by Appendix Eq. (3). In short, the hetero-functional adjacency matrix (or tensor) unambigously describes the sequence of two subject+verb+operand sentences whereas neither of the above interpretations of a multi-layer network do.

### Network descriptors

In light of the commonalities and differences between hetero-functional graphs and (formal) multilayer networks, this section discusses the meaning of network descriptors in the context of hetero-functional graphs. In this regard, the hetero-functional adjacency matrix is an adjacency matrix like any other. Consequently, network descriptors can be calculated straightforwardly. Furthermore, network descriptors can be applied to subsets of the graph so as to conduct a layer-by-layer analysis. Nevertheless, that the nodes in a hetero-functional graph represent whole-sentence-capabilities means that network descriptors have the potential to provide new found meanings over formal graphs based on exclusively formal elements.

#### Degree centrality

Degree centrality measures the number of edges attached to a vertex. Since a hetero-functional graph is a directed graph, there is a need to distinguish between the in-degree centrality, which measures the number of edges going into vertex, and the out-degree centrality, which measures the number of edges going out of a vertex^100^. In the context of hetero-functional graph theory, the in-degree centrality of a vertex calculates the number of capabilities that potentially proceed the capability related to the vertex. The out-degree centrality calculates the number of capabilities that potentially succeed the vertex’s capability. The higher the degree centrality of a capability, the more connected that capability is to the other capabilities in the hetero-functional graph. It is important to recognize that because transportation capabilities receive nodes in a hetero-functional graph, they can become the most central node. In contrast, the degree centrality of a formal graph could not reach such a conclusion because the function of transportation is tied to formal edges rather than formal nodes.

#### Closeness centrality

Closeness centrality measures the average shortest path from one vertex to every other reachable vertex in the graph. In a hetero-functional graph, the meaning of closeness centrality shows how a disruption has the potential to propagate through the graph across all different types of operands^100^. This metric is especially valuable for the resilience studies of interdependent systems, where the propagation of disruption across multiple disciplines is often poorly understood.

#### Eigenvector centrality

Eigenvector centrality calculates the importance of a node relative to the other nodes in the network^126^. It also includes the eigenvector centrality of the node’s direct neighbors^14^. The eigenvector centrality is specifically designed for the weighting of the in-degree of nodes in a directed network. The *Katz centrality*, on the other hand, provides an approach to study the relative importance of nodes based on the out-degree^14^.

#### Clustering coefficients

Clustering coefficients describe how strongly the nodes of a network cluster together. This is performed by searching for “triangles” or “circles” of nodes in a network. In a directed network, these circles can appear in multiple distinct combinations of directed connections. Each of these combinations needs to be measured and counted differently. Fagiolo discussed this taxonomy and accompanying clustering coefficients^127^. These clustering coefficients for directed networks can be directly applied to hetero-functional graphs and show which capabilities are strongly clustered together. The definition of layers in hetero-functional graphs allows for a consistent definition and calculation of clustering coefficients within and across layers for different types of systems. When investigating a system, the clustering coefficient may show clusters of capabilities that were not yet recognized as heavily interdependent. Such information can be used to revise control structures such that clusters of capabilities are controlled by the same entity for efficiency.

#### Modularity

Modularity serves as a measure to study if a network can be decomposed in disjoint sets. In the hetero-functional graph theory literature, much has been published about modularity as it was a prime motivation towards the inception of the theory^10,128^. Hetero-functional graph theory introduces the concept of the *Degree-of-Freedom-based Design Structure Matrix* (or: the capability DSM) that does not only encompass the hetero-functional adjacency matrix, but extends the concept to the other elements of hetero-functional graph theory: the service model and the control model. The hetero-functional graph design structure matrix has the ability to visualize the couplings between the subsystems of an engineering system and to classify those interfaces. Note that the capability DSM can also be applied to just the hetero-functional adjacency matrix. Furthermore, the capability DSM applies to the concept of layers in a hetero-functional graph. To study the interfaces between layers, the capability DSM can adopt layers as subsystems and classify the interfaces between the layers as mentioned previously. In conclusion, hetero-functional graphs are described by flat adjacency matrices, regardless of the number of layers in the analysis. Consequently, conventional graph theoretic network descriptors can be applied. The main difference in definition between the conventional graph theoretic application and the hetero-functional graph theoretic application is the result of the difference in the *definition of the fundamental modeling elements*, the nodes and edges, in a hetero-functional graph.

## Conclusions and future work

This paper has provided a tensor-based formulation of several of the most important parts of hetero-functional graph theory. More specifically, it discussed the system concept showing it as a generalization of formal graphs and multi-commodity networks. It also discussed the hetero-functional adjacency matrix and its tensor-based closed form calculation. It also discussed the hetero-functional incidence tensor and related it back to the hetero-functional adjacency matrix. The tensor-based formulation described in this work makes a stronger tie between HFGT and its ontological foundations in MBSE. Finally, the tensor-based formulation facilitates an understanding of the relationships between HFGT and multi-layer networks “despite its disparate terminology and lack of consensus”. In so doing, this tensor-based treatment is likely to advance Kivela et. al’s goal to discern the similarities and differences between these mathematical models in as precise a manner as possible.

## Supplementary Information

Supplementary Information.

## Abbreviations

$DOF_{\rho }$: Sequence-dependent degrees of freedom
${\mathbb {P}}_S$: A (non-unique) projection matrix for the vectorized knowledge base
${\widetilde{A}}_{\rho }$: Hetero-functional Adjacency Matrix after elimination of row and column sparsity
$\psi$: Index of the elements in the set of structural degrees of freedom ${{\mathcal {E}}}_S$
