# Supplementary material for: A tensor-based formulation of hetero-functional graph theory
Source: Sci Rep. 2022 Nov 5;12:18805. doi: 10.1038/s41598-022-19333-y (PMC9637230; doi:10.1038/s41598-022-19333-y)
Supplement: Supplementary file 1 — Supplementary Information. [file 41598_2022_19333_MOESM1_ESM.pdf]

Appendix

A. Ontological Science Definitions

The formal definitions of soundness, completeness, lucidity, and laconicity rely on "Ullman's Triangle" in Figure 1.

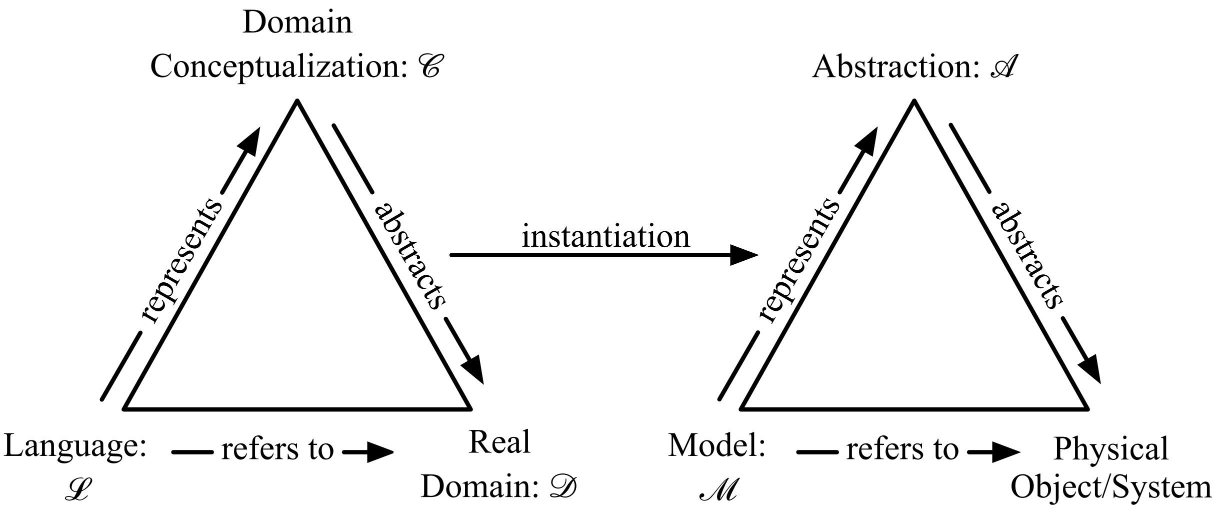

Fig. 1. Two Versions of Ullman's Triangle. On the left is the relationship between reality, the understanding of reality, and the description of reality. On the right the instantiated version of the ontological definition [1].

Definition 1 – Soundness [2]**:** A language $\mathcal{L}$ is sound w.r.t. a domain conceptualization $\mathcal{C}$ iff every modeling primitive in the language ($\mathcal{M}$) has an interpretation in the domain abstraction $\mathcal{A}$. (The absence of soundness results in the excess of modeling primitives w.r.t. the domain abstractions as shown in Figure 2.c on lucidity.) ■

Definition 2 – Completeness [2]**:** A language $\mathcal{L}$ is complete w.r.t. a domain conceptualization $\mathcal{C}$ iff every concept in the domain abstraction $\mathcal{A}$ of that domain is represented in a modeling primitive of that language. (The absence of completeness results in one or more concepts in the domain abstraction not being represented by a modeling primitive, as shown in Figure 2.d on laconicity.) ■

Definition 3 – Lucidity [2]**:** A language $\mathcal{L}$ is lucid w.r.t. a domain conceptualization $\mathcal{C}$ iff every modeling primitive in the language represents at most one domain concept in abstraction $\mathcal{A}$. (The absence of lucidity results in the overload of a modeling primitive w.r.t. two or more domain concepts as shown in Figure 2.a on soundness.) ■

Definition 4 – Laconicity [2]**:** A language $\mathcal{L}$ is laconic w.r.t. a domain conceptualization $\mathcal{C}$ iff every concept in the abstraction $\mathcal{A}$ of that domain is represented at most once in the model of that language. (The absence of laconicity results in the redundancy of modeling primitives w.r.t the domain abstractions as shown in Figure 2.b on completeness.) ■

B. Notation Conventions

Several notation conventions are used throughout this work:

- All sets are indicated by a capital letter. e.g. P – the set of processes..
- All elements within a set are indicated by a lower case letter. e.g. $p \in P$.
- A subscript number indicates the position in an ordered set. e.g. $p_i \in P$.
- The ith elementary basis vector of size n is denoted by e_i^n.
- A matrix of ones of size $m \times n$ is denoted by $\mathbb{1}^{m \times n}$.
- A matrix of zeros of size $m \times n$ is denoted by $\mathbf{0}^{m \times n}$.
- With the exception of elementary basis vectors, all vectors and matrices are indicated with a capital letter. e.g. J_H.
- All tensors are indicated with capital letters in calligraphic script. e.g. $\mathcal{J}_\mathcal{H}$.

- All elements in vectors, matrices, and tensors are indicated with indices within parentheses. e.g. $J_S(w,v)$.
- A(:,i) denotes the ith column of A or equivalently the ith mode-1 fiber. The : indicates all elements of the vector.
- A(i,:) denotes the ith row of A or equivalently the ith mode-2 fiber.
- A(i,j,:) denotes the i,j mode-3 fiber of $\mathcal{A}$.
- Given the presence of Booleans, real numbers and their operators, this work refrains from the use of Einstein's (shorthand) tensor notation where the sigma-notation $\sum$ is eliminated.

C. Hetero-functional Graph Theory Definitions

Definition 5 – Transformation Resource [3]: A resource $r \in R$ is a transformation resource $m \in M$ iff it is capable of one or more transformation processes on one or more operands and it exists at a unique location in space. ■

Definition 6 – Independent Buffer [3]: A resource $r \in R$ is an independent buffer $b \in B$ iff it is capable of storing one or more operands and is not able to transform them or transport them to another location and it exists at a unique location in space. ■

Definition 7 – Transportation Resource [3]: A resource $r \in R$ is a transportation resource $h \in H$ iff it is capable of transporting one or more operands between an origin and a distinct destination, without transforming these operands. ■

Definition 8 – Buffer [3]: A resource $r \in R$ is a buffer $b_s \in B_S$ iff it is capable of storing one or more operands at a unique location in space. $B_S = M \cup B$. ■

Definition 9 – Transformation Process [3]: A process is a transformation process $p_{\mu j} \in P_\mu$ iff it is capable of transforming one or more properties of a set of operands into a distinct set of output properties in place. It's syntax is:

$$\{\text{transitive verb, operands}\} \rightarrow \{\text{outputs}\} \tag{1}$$

■

Definition 10 – Refined Transportation Process [3]: A process is a refined transportation process $p_{\bar{\eta}\varphi} \in P_{\bar{\eta}}$ iff it is capable of transporting one or more operands between an origin buffer $b_{sy_1} \in B_S$ to a destination buffer $b_{sy_2} \in B_S$ while it is realizing holding process $p_{\gamma g} \in P_\gamma$. It's syntax is:

$$\{\text{transport, operands, origin, destination,while transitive verb}\} \rightarrow \{\text{outputs, destination}\} \tag{2}$$

■

Definition 11 – Transportation Process [3]: A process is a transportation process $p_{\eta u} \in P_\eta$ iff it is capable of transporting one or more operands between an origin buffer $b_{sy_1} \in B_S$ to a destination buffer $b_{sy_2} \in B_S$ according to the following convention of indices [4]–[8][1]:

$$u = \sigma(B_S)(y_1 - 1) + y_2 \tag{3}$$

It's syntax is:

$$\{\text{transport, operands,origin,destination}\} \rightarrow \{\text{outputs,destination}\} \tag{4}$$

■

Definition 12 – Holding Process [3]: A process is a holding process $p_{\gamma g} \in P_\gamma$ iff it holds one or more operands during the transportation from one buffer to another. In order to maintain the independence axiom and the mutual exclusivity of the system processes (Theorem 1), holding processes are specified so as to distinguish between transportation processes that:

- Have *different* operands,

[1]Note that a "storage process" is merely a transportation process with the same origin and destination.

- Hold a given operand in a *given way*, or
- Change the *state* of the operand.

■

Theorem 1 – Mutual Exclusivity of System Processes [3]: A lucid representation of system processes as a domain conceptualization distinguishes between two system processes as modeling primitives with different sets of inputs and outputs. ■

Definition 13 – Capability [4]–[9]: An action $e_{wv} \in \mathcal{E}_S$ (in the SysML sense) defined by a system process $p_w \in P$ being executed by a resource $r_v \in R$. It constitutes a subject + verb + operand sentence of the form: "Resource r_v does process p_w". ■

Definition 14 – The Negative Transformation Process-Operand Incidence Matrix $M^-_{LP_\mu}$: A binary incidence matrix $M^-_{LP_\mu} \in \{0,1\}^{\sigma(L)\times\sigma(P_\mu)}$ whose element $M^-_{LP}(i,j) = 1$ when the transformation system process $p_{\mu_j} \in P$ pulls operand $l_i \in L$ as an input.

■

Definition 15 – The Negative Refined Transportation Process-Operand Incidence Matrix $M^-_{LP_{\bar\eta}}$: A binary incidence matrix $M^-_{LP_{\bar\eta}} \in \{0,1\}^{\sigma(L)\times\sigma(P_{\bar\eta})}$ whose element $M^-_{LP}(i,\varphi) = 1$ when the refined transportation process $p_\varphi \in P_{\bar\eta}$ pulls operand $l_i \in L$ as an input. It is calculated directly from the negative holding process-operand incidence matrix $M^-_{LP_\gamma}$.

$$M^-_{LP_{\bar\eta}}(i,\varphi) = \sum_{u=1}^{\sigma(P_\eta)} M^-_{LP_\gamma}(i,g)\cdot\delta_u \qquad \forall i \in \{1,\ldots\sigma(L)\},\ g \in \{1,\ldots,\sigma(P_\gamma)\}, u \in \{1,\ldots,\sigma(P_\eta)\}, \varphi = \sigma(P_\eta)(g-1)+u \tag{5}$$

$$M^-_{LP_{\bar\eta}}(i,\varphi) = \sum_{y_1=1}^{\sigma(B_S)}\sum_{y_2=1}^{\sigma(B_S)} M^-_{LP_\gamma}(i,g)\cdot\delta_{y_1}\cdot\delta_{y_2} \quad \forall y_1,y_2 \in \{1,\ldots,\sigma(B_S)\}, \varphi = \sigma^2(B_S)(g-1)+\sigma(B_S)(y_1-1)+y_2 \tag{6}$$

$$M^-_{LP_{\bar\eta}} = \sum_{u=1}^{\sigma(P_\eta)} M^-_{LP_\gamma}\otimes e_u^{\sigma(P_\eta)T} \qquad = \sum_{y_1=1}^{\sigma(B_S)}\sum_{y_2=1}^{\sigma(B_S)} M^-_{LP_\gamma}\otimes\left(e_{y_1}^{\sigma(B_S)}\otimes e_{y_2}^{\sigma(B_S)}\right)^T \tag{7}$$

$$= M^-_{LP_\gamma}\otimes \mathbb{1}^{\sigma(P_\eta)T} \qquad = M^-_{LP_\gamma}\otimes\left(\mathbb{1}^{\sigma(B_S)}\otimes\mathbb{1}^{\sigma(B_S)}\right)^T \tag{8}$$

■

Definition 16 – The Negative Holding Process-Operand Incidence Matrix $M^-_{LP_\gamma}$: A binary incidence matrix $M^-_{LP_\gamma} \in \{0,1\}^{\sigma(L)\times\sigma(P_\gamma)}$ whose element $M^-_{LP}(i,g) = 1$ when the holding process $p_g \in P_\gamma$ pulls operand $l_i \in L$ as an input.

■

Definition 17 – The Positive Transformation Process-Operand Incidence Matrix $M^+_{LP_\mu}$: A binary incidence matrix $M^+_{LP_\mu} \in \{0,1\}^{\sigma(L)\times\sigma(P_\mu)}$ whose element $M^+_{LP}(i,j) = 1$ when the transformation system process $p_{\mu_j} \in P$ ejects operand $l_i \in L$ as an output.

■

Definition 18 – The Positive Refined Transportation Process-Operand Incidence Matrix $M^+_{LP_{\bar\eta}}$: A binary incidence matrix $M^+_{LP_{\bar\eta}} \in \{0,1\}^{\sigma(L)\times\sigma(P_{\bar\eta})}$ whose element $M^+_{LP}(i,\varphi) = 1$ when the refined transportation process $p_\varphi \in P_{\bar\eta}$ ejects operand $l_i \in L$ as an output. It is calculated directly from the negative holding

process-operand incidence matrix $M^+_{LP_\gamma}$.

$$M^+_{LP_{\bar{\eta}}}(i,\varphi) = \sum_{u=1}^{\sigma(P_\eta)} M^+_{LP_\gamma}(i,g)\cdot\delta_u \qquad \forall i \in \{1,\dots\sigma(L)\},\ g\in\{1,\dots,\sigma(P_\gamma)\}, u\in\{1,\dots,\sigma(P_\eta)\}, \varphi = \sigma(P_\eta)(g-1)+u \tag{9}$$

$$M^+_{LP_{\bar{\eta}}}(i,\varphi) = \sum_{y_1=1}^{\sigma(B_S)}\sum_{y_2=1}^{\sigma(B_S)} M^+_{LP_\gamma}(i,g)\cdot\delta_{y_1}\cdot\delta_{y_2} \quad \forall y_1,y_2\in\{1,\dots,\sigma(B_S)\}, \varphi = \sigma^2(B_S)(g-1)+\sigma(B_S)(y_1-1)+y_2 \tag{10}$$

$$M^+_{LP_{\bar{\eta}}} = \sum_{u=1}^{\sigma(P_\eta)} M^+_{LP_\gamma}\otimes e_u^{\sigma(P_\eta)T} \qquad = \sum_{y_1=1}^{\sigma(B_S)}\sum_{y_2=1}^{\sigma(B_S)} M^+_{LP_\gamma}\otimes\left(e_{y_1}^{\sigma(B_S)}\otimes e_{y_2}^{\sigma(B_S)}\right)^T \tag{11}$$

$$= M^+_{LP_\gamma}\otimes \mathbb{1}^{\sigma(P_\eta)T} \qquad = M^+_{LP_\gamma}\otimes\left(\mathbb{1}^{\sigma(B_S)}\otimes\mathbb{1}^{\sigma(B_S)}\right)^T \tag{12}$$

■

Definition 19 – The Positive Holding Process-Operand Incidence Matrix $M^+_{LP_\gamma}$: A binary incidence matrix $M^+_{LP_\gamma}\in\{0,1\}^{\sigma(L)\times\sigma(P_\gamma)}$ whose element $M^+_{LP}(i,g)=1$ when the holding process $p_g\in P_\gamma$ ejects operand $l_i\in L$ as an output.

■

D. Definitions of Set Operations

Definition 20 – $\sigma()$ Notation [3]: returns the size of the set. Given a set S with n elements, $n=\sigma(S)$. ■

Definition 21 – Cartesian Product $\times$ [10]: Given three sets, A, B, and C,

$$A\times B = \{(a,b)\in C \quad \forall a\in A \text{ and } b\in B\} \tag{13}$$

■

E. Definitions of Boolean Operations

The conventional symbols of $\wedge$, $\vee$, and $\neg$ are used to indicate the AND, OR, and NOT operations respectively.

Definition 22 – $\bigvee$ Notation: $\bigvee$ notation indicates a Boolean OR over multiple binary elements a_i.

$$\bigvee_i^n a_i = a_1\vee a_2\vee\ldots\vee a_n \tag{14}$$

■

Definition 23 – Matrix Boolean Addition $\oplus$: Given Boolean matrices $A,B,C\in\{0,1\}^{m\times n}$, $C=A\oplus B$ is equivalent to

$$C(i,j) = A(i,j)\vee B(i,j) \qquad \forall i\in\{1\ldots m\}, j\in\{1\ldots n\} \tag{15}$$

■

Definition 24 – Matrix Boolean Scalar Multiplication $(\cdot)$: Given Boolean matrices $A,B,C\in\{0,1\}^{m\times n}$, $C=A\cdot B$ is equivalent to

$$C(i,j) = A(i,j)\wedge B(i,j) = A(i,j)\cdot B(i,j) \qquad \forall i\in\{1\ldots m\}, j\in\{1\ldots n\} \tag{16}$$

■

Definition 25 – Matrix Boolean Multiplication $\odot$ [5], [11]: Given matrices $A\in\{0,1\}^{m\times n}$, $B\in\{0,1\}^{n\times p}$, and $C\in\{0,1\}^{m\times p}$, $C=A\times B=AB$ is equivalent to

$$C(i,k) = \bigvee_{i=1}^n A(i,j)\wedge B(j,k) = \bigvee_{i=1}^n A(i,j)\cdot B(j,k) \qquad \forall i\in\{1\ldots m\}, k\in\{1\ldots p\} \tag{17}$$

■

Definition 26 – Matrix Boolean Subtraction: Given Boolean matrices $A, B, C \in \{0,1\}^{m\times n}$, $C = A \ominus B$ is equivalent to

$$C(i,k) = A(i,j) \wedge \neg B(i,j)) = A(i,j)\cdot \neg B(i,j) \qquad \forall i \in \{1\dots m\}, j \in \{1\dots n\} \tag{18}$$

■

F. Matrix Operations

Definition 27 – Kronecker Delta Function δ_{ij} [12]:

$$\delta_{ij} = \begin{cases} 1 & \text{if } i = j \\ 0 & \text{if } i \neq j \end{cases} \tag{19}$$

■

Definition 28 – Hadamard Product [13]: Given matrices $A, B, C \in \mathbb{R}^{m\times n}$, $C = A \cdot B$ is equivalent to

$$C(i,j) = A(i,j)\cdot B(i,j) \qquad \forall i \in \{1\dots m\}, j \in \{1\dots n\} \tag{20}$$

■

Definition 29 – Matrix Product [13]: Given matrices $A \in \mathbb{R}^{m\times n}$, $B \in \mathbb{R}^{n\times p}$, and $C \in \mathbb{R}^{m\times p}$, $C = A \times B = AB$ is equivalent to

$$C(i,k) = \sum_{i=1}^{n} A(i,j)\cdot B(j,k) \qquad \forall i \in \{1\dots m\}, k \in \{1\dots p\} \tag{21}$$

■

Definition 30 – Kronecker Product [14], [15]: Given matrix $A \in \mathbb{R}^{m\times n}$ and $B \in \mathbb{R}^{p\times q}$, the Kronecker (kron) product denoted by $C = A \otimes B$ is given by:

$$C = \begin{bmatrix} A(1,1)B & A(1,2)B & \dots & A(1,n)B \\ A(2,1)B & A(2,2)B & \dots & A(2,n)B \\ \vdots & \vdots & \ddots & \vdots \\ A(m,1)B & A(m,2)B & \dots & A(m,n)B \end{bmatrix} \tag{22}$$

Alternatively, in scalar notation:

$$C(p(i-1)+k, q(j-1)+l) = a(i,j)\cdot b(k,l) \qquad \forall i \in \{1\dots m\}, j \in \{1\dots n\}, k \in \{1\dots p\}, l \in \{1\dots q\} \tag{23}$$

■

Definition 31 – Khatri-Rao Product [14], [15]: The Khatri-Rao Product is the “column-wise Kronecker product”. Given matrix $A \in \mathbb{R}^{m\times n}$ and $B \in \mathbb{R}^{p\times n}$, the Khatri-Rao product denoted by $C = A \circledast B$ is given by:

$$C = \begin{bmatrix} A(:,1)\otimes B(:,1) & A(:,2)\otimes B(:,2) & \dots & A(:,n)\otimes B(:,n) \end{bmatrix} \tag{24}$$

$$= [A \otimes \mathbb{1}^{p}]\cdot[\mathbb{1}^{m} \otimes B] \tag{25}$$

Alternatively, in scalar notation:

$$C(p(i-1)+k, j) = a(i,j)\cdot b(k,j) \qquad \forall i \in \{1\dots m\}, j \in \{1\dots n\}, k \in \{1\dots p\} \tag{26}$$

If A and B are column vectors, the Kronecker and Khatri-Rao products are identical. ■

G. Tensor Operations

Definition 32 – Outer Product of Vectors [14], [15]: Given two vectors $A_1 \in \mathbb{R}^{m_1}$ and $A_2 \in \mathbb{R}^{m_2}$, their outer product $B \in \mathbb{R}^{m_1 \times m_2}$ is denoted by

$$B = A_1 \circ A_2 = A_1 A_2^T \tag{27}$$

$$B(i_1, i_2) = A_1(i_1) \cdot A_2(i_2) \qquad \forall i_1 \in \{1 \ldots m_1\}, i_2 \in \{1 \ldots m_2\} \tag{28}$$

Given n vectors, $A_1 \in \mathbb{R}^{m_1}, A_2 \in \mathbb{R}^{m_2}, \ldots, A_n \in \mathbb{R}^{m_n}$, their outer product $\mathcal{B} \in \mathbb{R}^{m_1 \times m_2 \times \ldots \times m_n}$ is denoted by $\mathcal{B} = A_1 \circ A_2 \circ \ldots \circ A_n$ where

$$\mathcal{B}(i_1, i_2, \ldots, i_n) = A_1(i_1) \cdot A_2(i_2) \cdot \ldots \cdot A_n(i_n) \quad \forall i_1 \in \{1 \ldots m_1\}, i_2 \in \{1 \ldots m_2\}, \ldots, i_n = \{1 \ldots m_n\} \tag{29}$$

■

Definition 33 – Matricization $\mathcal{F}_M$([14], [15]): Given an nth order tensor $\mathcal{A} \in \mathbb{R}^{p_1 \times p_2 \times \ldots \times p_n}$, and ordered sets $R = \{r_1, \ldots, r_L\}$ and $C = \{c_1, \ldots, c_M\}$ that are a partition of the n modes $N = \{1, \ldots, n\}$ (i.e. $R \cup C = N, R \cap C = \emptyset$), the matricization function $\mathcal{F}_M()$ outputs the matrix $A \in \mathbb{R}^{J \times K}$

$$A = \mathcal{F}_M(\mathcal{A}, R, C) \tag{30}$$

$$A(j,k) = \mathcal{A}(i_1, i_2, \ldots, i_n) \qquad \forall i_1 \in \{1, \ldots, p_1\}, i_2 \in \{1, \ldots, p_2\}, \ldots i_n \in \{1, \ldots, p_n\} \tag{31}$$

where

$$j = 1 + \sum_{l=1}^{L} \left[(i_{r_l} - 1) \prod_{l'=1}^{l-1} i_{r_{l'}} \right], \quad k = 1 + \sum_{m=1}^{M} \left[(i_{c_m} - 1) \prod_{m'=1}^{m-1} i_{c_{m'}} \right], \quad J = \prod_{q \in R} p_q \quad K = \prod_{q \in C} p_q \tag{32}$$

For the sake of clarity, $\mathcal{F}_M()$ is implemented in MATLAB code:

```
ATensor = rand(4,7,5,3); R = [4 1]; C = [2 3];
function AMatrix=matricize(ATensor,R,C);
P = size(ATensor); J = prod(P(R)); K = prod(P(C));
AMatrix = reshape(permute(ATensor,[R C]),J,K); % Matricize
```

■

Definition 34 – Tensorization [14], [15]: Given a matrix $A \in \mathbb{R}^{J \times K}$, the dimensions $P = [p_1, p_2, \ldots, p_n]$ of a target nth order tensor $\mathcal{A} \in \mathbb{R}^{p_1 \times p_2 \times \ldots \times p_n}$, and ordered sets $R = \{r_1, \ldots, r_L\}$ and $C = \{c_1, \ldots, c_M\}$ that are a partition of the n modes $N = \{1, \ldots, n\}$ (i.e. $R \cup C = N, R \cap C = \emptyset$), the tensorization function $\mathcal{F}_M^{-1}()$ outputs the nth order tensor $\mathcal{A}$.

$$\mathcal{A} = \mathcal{F}_M^{-1}(A, P, R, C) \tag{33}$$

$$\mathcal{A}(i_1, i_2, \ldots, i_n) = A(j,k) \qquad \forall i_1 \in \{1, \ldots, p_1\}, i_2 \in \{1, \ldots, p_2\}, \ldots i_n \in \{1, \ldots, p_n\} \tag{34}$$

where

$$j = 1 + \sum_{l=1}^{L} \left[(i_{r_l} - 1) \prod_{l'=1}^{l-1} i_{r_{l'}} \right], \quad k = 1 + \sum_{m=1}^{M} \left[(i_{c_m} - 1) \prod_{m'=1}^{m-1} i_{c_{m'}} \right], \quad J = \prod_{q \in R} p_q \quad K = \prod_{q \in C} p_q \tag{35}$$

For the sake of clarity, $\mathcal{F}_M^{-1}()$ is implemented in MATLAB code:

```
AMatrix = rand(12,35); P=[4,7,5,3]; R = [4 1]; C = [2 3];
function ATensor=tensorize(AMatrix,P,R,C);
ATensor = ipermute(reshape(AMatrix,[P(R) P(C)]),[R C]); % Tensorize
```

■

Definition 35 – Vectorization [13]–[15]: Vectorization denoted by $vec()$ or $()^V$ as a shorthand is a special case of matricization when the resulting matrix is simply a vector. Formally, given an nth order tensor

$\mathcal{A} \in \mathbb{R}^{p_1 \times p_2 \times \ldots \times p_n}$ and the dimensions $P = [p_1, p_2, \ldots, p_n]$, the vectorization function $vec() = ()^V$ outputs the vector $A \in \mathbb{R}^J$

$$A = vec(\mathcal{A}) = \mathcal{A}^V \tag{36}$$

$$A(j) = \mathcal{A}(i_1, i_2, \ldots, i_n) \qquad \forall i_1 \in \{1, \ldots, p_1\}, i_2 \in \{1, \ldots, p_2\}, \ldots i_n \in \{1, \ldots, p_n\} \tag{37}$$

where

$$j = 1 + \sum_{l=1}^{n} \left[(i_l - 1) \prod_{l'=1}^{l-1} i_{l'} \right], \quad J = \prod_{q=1}^{n} p_q \tag{38}$$

■

Definition 36 – Inverse Vectorization [13]–[15]: Inverse vectorization denoted by $vec^{-1}()$ is a special case of tensorization when the input matrix is simply a vector. Formally, $A \in \mathbb{R}^J$ and the dimensions $P = [p_1, p_2, \ldots, p_n]$ of a target nth order tensor $\mathcal{A} \in \mathbb{R}^{p_1 \times p_2 \times \ldots \times p_n}$, the inverse vectorization function $vec^{-1}()$ outputs the nth order tensor $\mathcal{A}$.

$$\mathcal{A} = vec^{-1}(A, P) \tag{39}$$

$$\mathcal{A}(i_1, i_2, \ldots, i_n) = A(j) \qquad \forall i_1 \in \{1, \ldots, p_1\}, i_2 \in \{1, \ldots, p_2\}, \ldots i_n \in \{1, \ldots, p_n\} \tag{40}$$

where

$$j = 1 + \sum_{l=1}^{n} \left[(i_l - 1) \prod_{l'=1}^{l-1} i_{l'} \right], \quad J = \prod_{q=1}^{n} p_q \tag{41}$$

Furthermore, the above definition of inverse vectorization can be applied to a qth dimensional slice of a tensor. In such a case,

$$\mathcal{B} = vec^{-1}(A, P, r) \tag{42}$$

$$\mathcal{B}(k_1, \ldots, k_{r-1}, i_1, \ldots, i_n, k_{r+1}, \ldots, k_m) = \mathcal{A}(k_1, \ldots, k_{r-1}, j, k_{r+1}, \ldots, k_m) \tag{43}$$

where index convention in Equation 41 applies. ■

Definition 37 – Matrix and Tensor Transpose: Given a matrix $A \in \mathbb{R}^{m_1 \times m_2}$, its matrix transpose $A^T \in \mathbb{R}^{m_2 \times m_1}$ is equivalent to:

$$A^T(j, i) = A(i, j) \qquad \forall i \in \{1 \ldots m_1\}, j \in \{1 \ldots m_2\} \tag{44}$$

In this work, the generalization to tensors is a special case of the definition provided in [16]. Given a tensor $\mathcal{A} \in \mathbb{R}^{m_1 \times \ldots \times m_n}$, its tensor transpose $\mathcal{A}^T \in \mathbb{R}^{m_n \times \ldots \times m_1}$ is equivalent to:

$$\mathcal{A}^T(i_n, \ldots, i_1) = \mathcal{A}(i_1, \ldots i_n) \qquad \forall i_1 \in \{1 \ldots m_1\}, \ldots, i_n \in \{1 \ldots m_n\} \tag{45}$$

■

Definition 38 – N-Mode Matrix Product $\times_p$ [14], [15]: The N-mode matrix product is a generalization of the matrix product. Given a tensor $\mathcal{A} \in \mathbb{R}^{m_1 \times m_2 \times \ldots \times m_p \times \ldots \times m_n}$, matrix $B \in \mathbb{R}^{m_p \times q}$, and $\mathcal{C} \in \mathbb{R}^{m_1 \times m_2 \times \ldots \times q \times \ldots \times m_n}$, the n-mode matrix product denoted by $\mathcal{C} = \mathcal{A} \times_p B$ is equivalent to:

$$\mathcal{C}(i_1, i_2, \ldots, i_{p-1}, j, i_{p+1}, \ldots, i_n) = \sum_{i_p=1}^{m_p} \mathcal{A}(i_1, i_2, \ldots, i_n) \cdot B(i_p, j) \tag{46}$$

$$\forall i_1 \in \{1, \ldots, m_1\}, \ldots, i_{p-1} \in \{1, \ldots, m_{p-1}\}, i_{p+1} \in \{1, \ldots, m_{p+1}\}, \ldots, i_n \in \{1, \ldots, m_n\}, j \in \{1, \ldots, q\}$$

■

Definition 39 – N-Mode Boolean Matrix Product: The N-mode Boolean matrix product is a generalization of the Boolean matrix product. Given a tensor $\mathcal{A} \in \{0,1\}^{m_1 \times m_2 \times \ldots \times m_p \times \ldots \times m_n}$, matrix $B \in \{0,1\}^{m_p \times q}$, and $\mathcal{C} \in \{0,1\}^{m_1 \times m_2 \times \ldots \times q \times \ldots \times m_n}$, the n-mode matrix product denoted by $\mathcal{C} = \mathcal{A} \odot_p B$ is equivalent to:

$$\mathcal{C}(i_1, i_2, \ldots, i_{p-1}, j, i_{p+1}, \ldots, i_n) = \bigvee_{i_p=1}^{m_p} \mathcal{A}(i_1, i_2, \ldots, i_n) \cdot B(i_p, j) \tag{47}$$

$$\forall i_1 \in \{1, \ldots, m_1\}, \ldots, i_{p-1} \in \{1, \ldots, m_{p-1}\}, i_{p+1} \in \{1, \ldots, m_{p+1}\}, \ldots, i_n \in \{1, \ldots, m_n\}, j \in \{1, \ldots, q\}$$

■

References

[1] G. Guizzardi, "On ontology, ontologies, conceptualizations, modeling languages, and (meta) models," *Frontiers in artificial intelligence and applications*, vol. 155, p. 18, 2007.

[2] ——, *Ontological foundations for structural conceptual models.* CTIT, Centre for Telematics and Information Technology, 2005.

[3] W. C. Schoonenberg, I. S. Khayal, and A. M. Farid, *A Hetero-functional Graph Theory for Modeling Interdependent Smart City Infrastructure.* Berlin, Heidelberg: Springer, 2019. [Online]. Available: http://dx.doi.org/10.1007/978-3-319-99301-0

[4] A. M. Farid and D. C. McFarlane, "A Development of Degrees of Freedom for Manufacturing Systems," in *IMS'2006: 5th International Symposium on Intelligent Manufacturing Systems: Agents and Virtual Worlds*, Sakarya, Turkey, 2006, pp. 1–6. [Online]. Available: http://engineering.dartmouth.edu/liines/resources/Conferences/IEM-C02.pdf

[5] A. M. Farid, "Reconfigurability Measurement in Automated Manufacturing Systems," Ph.D. Dissertation, University of Cambridge Engineering Department Institute for Manufacturing, 2007. [Online]. Available: http://engineering.dartmouth.edu/liines/resources/Theses/IEM-TP00.pdf

[6] A. M. Farid and D. C. McFarlane, "Production degrees of freedom as manufacturing system reconfiguration potential measures," *Proceedings of the Institution of Mechanical Engineers, Part B (Journal of Engineering Manufacture) – invited paper*, vol. 222, no. B10, pp. 1301–1314, 2008. [Online]. Available: http://dx.doi.org/10.1243/09544054JEM1056

[7] A. M. Farid, "Product Degrees of Freedom as Manufacturing System Reconfiguration Potential Measures," *International Transactions on Systems Science and Applications – invited paper*, vol. 4, no. 3, pp. 227–242, 2008. [Online]. Available: http://engineering.dartmouth.edu/liines/resources/Journals/IEM-J04.pdf

[8] ——, "Static Resilience of Large Flexible Engineering Systems: Axiomatic Design Model and Measures," *IEEE Systems Journal*, vol. PP, no. 99, pp. 1–12, 2015. [Online]. Available: http://dx.doi.org/10.1109/JSYST.2015.2428284

[9] ——, "An engineering systems introduction to axiomatic design," in *Axiomatic Design in Large Systems: Complex Products, Buildings & Manufacturing Systems*, A. M. Farid and N. P. Suh, Eds. Berlin, Heidelberg: Springer, 2016, ch. 1, pp. 1–47. [Online]. Available: http://dx.doi.org/10.1007/978-3-319-32388-6

[10] Anonymous, "Cartesian product," Wikipedia, Tech. Rep., 2021. [Online]. Available: https://en.wikipedia.org/wiki/Cartesian_product#:~:text=In%20mathematics%2C%20specifically%20set%20theory,and%20a%20set%20of%20columns.

[11] M. J. Fischer and A. R. Meyer, "Boolean matrix multiplication and transitive closure," in *Switching and Automata Theory, 1971., 12th Annual Symposium on.* IEEE, 1971, pp. 129–131.

[12] Anonymous, "Kronecker delta," Wikipedia, Tech. Rep., 2021. [Online]. Available: https://en.wikipedia.org/wiki/Kronecker_delta

[13] G. Golub and C. Van Loan, *Matrix Computations.* Johns Hopkins University Press, 1996.

[14] T. G. Kolda and B. W. Bader, "Tensor decompositions and applications," *SIAM review*, vol. 51, no. 3, pp. 455–500, 2009.

[15] T. G. Kolda, "Multilinear operators for higher-order decompositions." Sandia National Laboratories, Tech. Rep., 2006.

[16] R. Pan, "Tensor transpose and its properties," *arXiv preprint arXiv:1411.1503*, 2014.
